# Similar factors underlie tree abundance in forests in native and alien ranges

**DOI:** 10.1111/geb.13027

**Published:** 2019-12-01

**Authors:** Masha T. van der Sande, Helge Bruelheide, Wayne Dawson, Jürgen Dengler, Franz Essl, Richard Field, Sylvia Haider, Mark van Kleunen, Holger Kreft, Joern Pagel, Jan Pergl, Oliver Purschke, Petr Pyšek, Patrick Weigelt, Marten Winter, Fabio Attorre, Isabelle Aubin, Erwin Bergmeier, Milan Chytrý, Matteo Dainese, Michele De Sanctis, Jaime Fagundez, Valentin Golub, Greg R. Guerin, Alvaro G. Gutiérrez, Ute Jandt, Florian Jansen, Borja Jiménez‐Alfaro, Jens Kattge, Elizabeth Kearsley, Stefan Klotz, Koen Kramer, Marco Moretti, Ülo Niinemets, Robert K. Peet, Josep Penuelas, Petr Petřík, Peter B. Reich, Brody Sandel, Marco Schmidt, Maria Sibikova, Cyrille Violle, Timothy J. S. Whitfeld, Thomas Wohlgemuth, Tiffany M. Knight

**Affiliations:** ^1^ Department of Community Ecology Helmholtz Centre for Environmental Research–UFZ Halle (Saale) Germany; ^2^ German Centre for Integrative Biodiversity Research (iDiv) Halle‐Jena‐Leipzig Leipzig Germany; ^3^ Department of Biological Sciences Florida Institute of Technology Melbourne Florida; ^4^ Institute for Biodiversity & Ecosystem Dynamics University of Amsterdam Amsterdam The Netherlands; ^5^ Forest Ecology and Forest Management Group Wageningen University & Research Wageningen The Netherlands; ^6^ Martin Luther University Halle‐Wittenberg Institute of Biology/Geobotany and Botanical Garden Halle (Saale) Germany; ^7^ Department of Biosciences Durham University Durham United Kingdom; ^8^ Plant Ecology, Bayreuth Center of Ecology and Environmental Research (BayCEER), University of Bayreuth Bayreuth Germany; ^9^ Vegetation Ecology Institute of Environment and Natural Resources (IUNR), Zurich University of Applied Sciences (ZHAW) Switzerland; ^10^ Division of Conservation Biology, Vegetation Ecology and Landscape Ecology, Department of Botany and Biodiversity Research University of Vienna Vienna Austria; ^11^ School of Geography University of Nottingham Nottingham United Kingdom; ^12^ Ecology, Department of Biology University of Konstanz Konstanz Germany; ^13^ Zhejiang Provincial Key Laboratory of Plant Evolutionary Ecology and Conservation Taizhou University Taizhou China; ^14^ Biodiversity, Macroecology & Biogeography University of Goettingen Göttingen Germany; ^15^ Centre of Biodiversity and Sustainable Land Use (CBL), University of Goettingen Göttingen Germany; ^16^ Landscape & Plant Ecology University of Hohenheim Stuttgart Germany; ^17^ Institute of Botany Czech Academy of Sciences Průhonice Czech Republic; ^18^ Faculty of Science, Department of Ecology Charles University Prague Czech Republic; ^19^ Department of Environmental Biology University Sapienza of Rome Rome Italy; ^20^ Great Lakes Forestry Centre, Canadian Forest Service Natural Resources Canada Sault Ste Marie Ontario Canada; ^21^ Vegetation & Phytodiversity Analysis University of Göttingen Göttingen Germany; ^22^ Department of Botany and Zoology Masaryk University Brno Czech Republic; ^23^ Department of Animal Ecology and Tropical Biology, Biocenter University of Würzburg Würzburg Germany; ^24^ Institute for Alpine Environment EURAC Research Bolzano Italy; ^25^ Faculty of Science, Department of Biology University of A Coruña Coruña Spain; ^26^ Institute of Ecology of the Volga River Basin Russian Academy of Sciences Tolyatti Russia; ^27^ Terrestrial Ecosystem Research Network, School of Biological Sciences The University of Adelaide Adelaide South Australia Australia; ^28^ Departamento de Ciencias Ambientales y Recursos Naturales Renovables, Facultad de Ciencias Agronómicas Universidad de Chile Santiago Chile; ^29^ Faculty of Agricultural and Environmental Science University of Rostock Rostock Germany; ^30^ Research Unit of Biodiversity (CSIC/UO/PA) University of Oviedo Mieres Spain; ^31^ Max Planck Institute for Biogeochemistry Jena Germany; ^32^ Computational and Applied Vegetation Ecology (CAVElab) Ghent University Ghent Belgium; ^33^ Vegetation, Forest and Landscape Ecology, Wageningen Environmental Research (Alterra) Wageningen University and Research Wageningen The Netherlands; ^34^ Swiss Federal Research Institute WSL, Biodiversity and Conservation Biology Birmensdorf Switzerland; ^35^ Chair of Crop Science and Plant Biology Estonian University of Life Sciences Tartu Estonia; ^36^ Estonian Academy of Sciences Tallinn Estonia; ^37^ Department of Biology University of North Carolina Chapel Hill North Carolina; ^38^ CSIC, Global Ecology Unit CREAF‐CSIC‐UAB Barcelona Spain; ^39^ CREAF Barcelona Spain; ^40^ Department of Forest Resources University of Minnesota St. Paul Minnesota; ^41^ Hawkesbury Institute for the Environment Western Sydney University Penrith South DC New South Wales Australia; ^42^ Department of Biology Santa Clara University Santa Clara California; ^43^ Data and Modelling Centre Senckenberg Biodiversity and Climate Research Centre (SBiK‐F) Frankfurt am Main Germany; ^44^ Scientific Service Palmengarten der Stadt Frankfurt Frankfurt am Main Germany; ^45^ Institute of Botany, Plant Science and Biodiversity Center Slovak Academy of Sciences Bratislava Slovakia; ^46^ Centre d’Ecologie Fonctionnelle et Evolutive (UMR 5175) CNRS, Université Paul Valéry Montpellier, EPHE, Univ Montpellier Montpellier France; ^47^ Department of Ecology and Evolutionary Biology Brown University Providence Rhode Island; ^48^ Swiss Federal Institute for Forest, Snow and Landscape Research WSL Birmensdorf Switzerland

**Keywords:** abundance, dissimilarity, forest, functional traits, global, plant invasion, trees

## Abstract

**Aim:**

Alien plant species can cause severe ecological and economic problems, and therefore attract a lot of research interest in biogeography and related fields. To identify potential future invasive species, we need to better understand the mechanisms underlying the abundances of invasive tree species in their new ranges, and whether these mechanisms differ between their native and alien ranges. Here, we test two hypotheses: that greater relative abundance is promoted by (a) functional difference from locally co‐occurring trees, and (b) higher values than locally co‐occurring trees for traits linked to competitive ability.

**Location:**

Global.

**Time period:**

Recent.

**Major taxa studied:**

Trees.

**Methods:**

We combined three global plant databases: sPlot vegetation‐plot database, TRY plant trait database and Global Naturalized Alien Flora (GloNAF) database. We used a hierarchical Bayesian linear regression model to assess the factors associated with variation in local abundance, and how these relationships vary between native and alien ranges and depend on species’ traits.

**Results:**

In both ranges, species reach highest abundance if they are functionally similar to co‐occurring species, yet are taller and have higher seed mass and wood density than co‐occurring species.

**Main conclusions:**

Our results suggest that light limitation leads to strong environmental and biotic filtering, and that it is advantageous to be taller and have denser wood. The striking similarities in abundance between native and alien ranges imply that information from tree species’ native ranges can be used to predict in which habitats introduced species may become dominant.

## 1 INTRODUCTION

Biological invasions by alien species can cause severe environmental and economic problems (Mack et al., 2000; Petit, Bialozyt, Garnier‐Géré & Hampe, 2004; Richardson, Hui, Nuñez & Pauchard, 2014; Vilà et al., 2010). Most alien species, however, are non‐invasive in their alien range (Pyšek et al., 2017; Williamson & Fitter, 1996), suggesting that species‐specific properties, such as adaptive traits or phylogenetic relationships, determine invasion potential. Moreover, many invasive alien species are not considered harmful in their native range (Colautti et al., 2014; Parker et al., 2013), which suggests that they can interact in novel ways with the species in the new conditions of their alien range. One way to assess a species’ success in its native and alien ranges is its abundance relative to co‐occurring native species (Van Couwenberghe, Collet, Pierrat, Verheyen & Gégout, 2013). Surprisingly few studies have compared abundance differences in the native versus alien ranges of plant species (Firn et al., 2011; Gallien & Carboni, 2017; Hierro, Maron & Callaway, 2005; van Kleunen, Dawson, Schlaepfer, Jeschke & Fischer, 2010), and the ones that did mainly focused on the enemy‐release hypothesis (Colautti, Ricciardi, Grigorovich & MacIsaac, 2004) or on a small set of species (Callaway & Aschehoug, 2000; Taylor et al., 2016). A better global‐scale understanding of how patterns associated with mechanisms of coexistence differ between the native and alien ranges of species would provide insights into the invasion process that could help with the management of incipient invasions and the prevention of new ones.

The ability of alien species to establish and become dominant outside their native ranges can be driven by several mechanisms, such as favourable environmental conditions (Broennimann et al., 2007), novel properties of the alien species (Carboni et al., 2016) that lead to reduced competition via “niche differentiation” and/or release from enemies, more competitive traits (Lai, Mayfield, Gay‐des‐combes, Spiegelberger & Dwyer, 2015), and/or high propagule pressure due to active planting for ornamental or economic purposes (Conedera, Wohlgemuth, Tanadini & Pezzatti, 2018; Lockwood, Cassey & Blackburn, 2005; Pysek et al., 2015; Taylor et al., 2016). At local spatial scales (e.g., the community level), abundant alien species are often found to be phylogenetically and functionally more dissimilar to the native species than at broader spatial scales (Carboni et al., 2013), suggesting that niche differentiation increases alien success at local scales. However, high trait similarity among alien and native species can also occur if environmental conditions are such that only species with particular traits can survive, and/or when particular traits are associated with a competitive advantage and result in the exclusion of species with different traits (Kraft, Adler, et al., 2015). Reflecting this dual influence of biotic and abiotic drivers, we henceforth refer to such promotion of trait similarity as “environmental‐biotic filtering”. In the native range, species could become dominant due to similar mechanisms related to environmental conditions, niche differentiation and/or competitive differences (Chesson, 2000). The question remains, however, how niche differentiation and competitive advantage affect species abundance in the native vs. alien ranges, and how they affect the abundances of invasive in relation to non‐invasive alien species.

The success of alien tree species in forests is thought to be mainly driven by conditions of the invaded site and factors related to their introduction history, such as time since introduction, reason for introduction and, especially, propagule pressure (Bucharova & van Kleunen, 2009; Donaldson et al., 2014; Feng & van Kleunen, 2016; Richardson et al., 2014). However, just like other species, alien species that are classified as highly invasive only become locally dominant in some areas and remain rare in others, suggesting that local factors, such as their dissimilarity to the resident community or their competitive trait differences, could strongly determine species abundance at local scales (Stohlgren, 1999). The lack of research on local, plot‐scale abundances across broad geographic extents means that this remains an open question.

**Figure 1:**
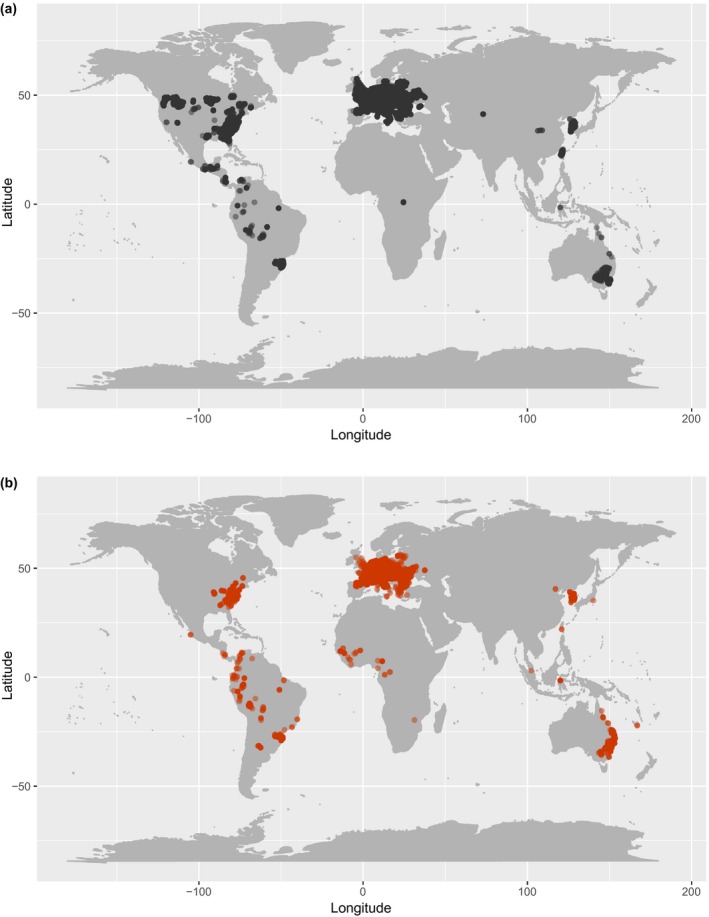
Plot locations in the native range (a) and alien range (b) across all 41 species. Points are transparent to show differences in plot densities [Colour figure can be viewed at https://www.wileyonlinelibrary.com]

Here, we evaluate the patterns of, and possible underlying factors associated with, the relative abundance (hereafter referred to as “abundance”) of tree species in forest ecosystems in their native and alien ranges. We focus on 41 tree species occurring in 228,943 plots around the world (Figure 1) that have their native and alien ranges on different continents. We address two research questions. First, how do species differ in their abundance between the native and alien ranges? We expect that invasive alien species are more (or equally) abundant in the alien compared to native range, whereas non‐invasive alien species are less (or equally) abundant in the alien compared to the native range. Second, how are the local abundances of tree species, in both their native and alien ranges, related to (multivariate) trait dissimilarity of co‐occurring woody species? We address this question in terms of both overall trait dissimilarity (as a measure of niche differentiation), and differences in traits specifically linked to competitive ability (see next paragraph). Furthermore, we examine the effects of human influence and climatic conditions on local abundance. We expected a priori that niche differentiation, competitive differences, and wet and warm climates would all enhance species abundance in both the native and the alien ranges. Human influence may favour alien species through transport, use and planting of these species in the surrounding human‐modified areas, which could increase propagule pressure in forests (i.e., spatial variation in how often species may arrive in the region), and through disturbances (e.g., gap openings) in forests.

We test two main hypotheses. First (hypothesis 1), in both the native and the alien ranges, local tree species abundance increases with high trait dissimilarity compared with co‐occurring woody species (Carboni et al., 2016). The importance of trait dissimilarity for abundance of species, however, may shift between the native and alien ranges, depending on environmental conditions. For example, if climatic conditions are more favourable in the alien range, then this could increase the importance of being functionally dissimilar to the co‐occurring species. Second (hypothesis 2), local abundance depends on competitive trait differences between focal and co‐occurring species: in this hypothesis, abundance is predicted to increase as values for traits related to competitive ability increase relative to co‐occurring species. Alien tree species that have higher growth and reproduction rates than their native competitors, and clonal ability (i.e., higher values for “competitive traits”), yet that have the capacity to invade dark forest understories, have been shown to be successful (Martin, Canham & Marks, 2009).

We focus on four traits that capture a large part of the functional differences among species with respect to competition for light and other resources, that are positioned along the *r–K* continuum and that are associated with tolerance to drought and shade (adult height, H; specific leaf area, SLA; seed mass, SM; wood density, WD; Westoby, 1998). All four traits have been reported to be good predictors of species' establishment, performance and abundance (Rejmanek & Richardson, 1996; Theoharides & Dukes, 2007; van Kleunen, Weber & Fischer, 2010), represent different tissues and ecological strategies (Westoby, 1998; Aubin et al., 2016), have high original coverage for our species (i.e., before gap‐filling), and are not strongly correlated (*r* < .4 in our data). H captures the trade‐off between a species’ capacity to benefit from high light levels and early reproduction (Westoby, 1998). SLA captures the trade‐off between light capture efficiency and strong, long‐lived leaves (Poorter, Niinemets, Poorter, Wright & Villar, 2009). SM increases recruitment in the understorey but usually at the cost of reduced seed number and, hence, colonization ability from seed rain (Bruun & Ten Brink, 2008; Rejmanek & Richardson, 1996; Thomson, Moles, Auld & Kingsford, 2011). WD is associated with high tolerance to shade and drought, and slow growth rates (Ameztegui et al., 2017; Markesteijn, Poorter, Bongers, Paz & Sack, 2011). For all four traits, higher values are taken to mean greater competitive advantage in these forest ecosystems. We address our questions and hypotheses by combining a global vegetation‐plot database (sPlot; Bruelheide et al., 2019), a global plant trait database (TRY; Kattge et al., 2011), and global information on naturalized alien plant species [Global Naturalized Alien Flora (GloNAF); van Kleunen et al., 2015].

## 2 METHODS

### 2.1 sPlot and GloNAF databases

We used the sPlot database, which contains vegetation plots across the globe in which composition and abundance of plant communities were recorded (http://www.idiv.de/splot; Bruelheide et al., 2019). To identify alien species in the plots, we matched the sPlot database with the GloNAF database (van Kleunen et al., 2019). We focused on neophytes, defined as species that were introduced after the year 1492 in the respective regions. The GloNAF database contains lists of alien species for non‐overlapping regions (countries or regions within countries) across the world. We could thus match all plots from sPlot with a region of GloNAF, and label the alien species in the plots. Species that were not labelled as “alien” were labelled as “native”.

### 2.2 Plot selection

We selected plots that had geographic coordinates because these could be linked to a GloNAF region, and that were classified as “forest” (i.e., with a minimum tree layer cover of 25%). It could be that some of the trees in the plots were planted. Unfortunately, we did not have detailed information about the land use history of the plots. To reduce the likelihood of including plantation forests, we excluded plots with fewer than three woody species. We also excluded plots that were smaller than 100 m^2^ because these often contain too few woody individuals for community‐level analyses. The average plot size was 362 m^2^, with a maximum of 40,000 m^2^ (4 ha). If species had plots in their alien range on the same continent as plots in their native range (e.g., some plots for *Acer negundo* and *Acer pseudoplatanus*), then these alien plots were excluded to ensure abiotic and biotic differences between the native and alien ranges. This resulted in native and alien ranges that were on different continents for all species. In total across the species, we used 228,943 plots, of which 213,359 were in the native ranges and 15,584 in the alien ranges (Figure 1).

### 2.3 Focal tree species selection

In total, we included 41 species from 24 families (Supporting Information Appendix S1). We included species that are part of the global tree list of Botanic Gardens Conservation International (http://www.bgci.org/global_tree_search.php), which also includes palms and some other tree‐like species. Our 41 “tree species” included 1 palm (*Syagrus romanzoffiana*), 1 non‐woody species (*Carica papaya*), 7 gymnosperm trees and 32 angiosperm trees. The focal tree species that we included occurred in at least three plots in their native and at least three in their alien range, and had their native and alien ranges on different continents. A total of 15 of these species are native to Eurasia, 24 to the Americas, 1 to Africa and 1 to Oceania. More alien tree species exist, but these were not found in sufficient numbers of plots and/or have their native and alien ranges on the same continent. The 41 alien species were classified as “invasive” or “non‐invasive” based on information from Rejmánek and Richardson (2013).

### 2.4 Species abundance

The “relative abundance” of a focal species per plot was calculated as the species’ share of the woody vegetation cover, based on canopy coverage. For all plots in sPlot, this relative cover of each species was calculated as the ratio between individual species' cover and total cover of all woody species. Across all plots, we had a total of 3,240 woody species. We excluded all non‐woody species from the calculation of relative coverage, so that we could test clear hypotheses among woody species in forest ecosystems.

### 2.5 Functional traits

We focused on specific leaf area (SLA; cm^2^/g), adult tree height (H; m), oven‐dried seed mass (SM; mg) and wood density (WD; g/cm^3^). We obtained trait information for all species in the study plots from the global trait database TRY (Kattge et al., 2011), by constructing a taxonomic backbone as described in Bruelheide et al. (2019). In sPlot, trait values for species without data in TRY were covered by gap‐filling using a Bayesian hierarchical probabilistic factorization (for details see Schrodt et al., 2015), which gave similar results to non‐gap‐filled data (Bruelheide et al., 2019). All traits are based on species‐level data; hence, we could not include intraspecific trait variation.

### 2.6 Dissimilarity and competitive trait differences

#### 2.6.1 Dissimilarity

As a measure of niche separation, we calculated multivariate trait dissimilarity (using SLA, H, SM and WD) between a focal tree species and co‐occurring woody species, for all plots in the native and alien ranges, using the multivariate trait dissimilarity index (Gower, 1971). High values of dissimilarity in these traits indicate weak environmental–biotic filtering. All trait values were log‐transformed before calculation of dissimilarity and competitive trait differences, to avoid strong effects of extreme values. The multivariate trait dissimilarity index is based on the absolute difference in trait values between the focal tree species and the abundance‐weighted mean of all co‐occurring woody species. We refer to this measure throughout the manuscript as “multivariate trait dissimilarity”. Because all of the traits are continuous, the multivariate trait dissimilarity index is based on range‐normalized Manhattan distances converted into similarity [which is correlated (*r* > .95) with dissimilarity based on Euclidean distances]. We calculated the multivariate trait dissimilarity index using the *daisy* function of the *cluster* package in R (Maechler, Rousseeuw, Struyf, Hubert & Hornik, 2017). We used multivariate trait dissimilarity instead of dissimilarity in single traits because these are more likely to capture niche differences (Kraft, Godoy, et al., 2015), to keep the model simpler and avoid collinearity among predictor variables, and to incorporate possible trade‐offs among traits. High values of trait dissimilarity indicate that the focal tree species is novel in terms of the measured traits compared to its co‐occurring species, whereas low values of trait dissimilarity indicate that the focal species is similar in these traits to its co‐occurring species.

#### 2.6.2 Competitive trait differences

To measure species’ competitive differences, we used the differences between ln‐transformed trait values of the focal tree species and the abundance‐weighted mean traits of the co‐occurring species. This was done separately for the four traits: SLA, H, SM and WD. Here, a positive value for any of the trait differences (ΔSLA, ΔH, ΔSM or ΔWD) in a plot indicates that the focal species has, on average, a higher respective trait value (i.e., more competitive with respect to that trait) than the co‐occurring woody species, whereas negative values indicate that the focal species has, on average, lower trait values (less competitive) than its neighbours.

For all indices, we used the abundance‐weighted mean distance instead of the minimal distance (i.e., the distance to the species in the plot that has most similar trait values), because abundance‐weighted indices are best at disentangling environmental filtering from competition effects (Gallien, Carboni & Münkemüller, 2014). If there were alien species in the plots in the native range, or alien species other than the focal tree species in the alien range, then these species were excluded. We kept all remaining angiosperm and gymnosperm woody species in the plots for the calculation of the above‐mentioned indices, as their trait values fall along similar ecological dimensions and are indicators of similar ecological functions (Díaz et al., 2015; Niklas & Spatz, 2010).

### 2.7 Human influence and climatic conditions

We used the human influence index (HII; Gallardo, Zieritz & Aldridge, 2015) as a proxy for human influence. The data were obtained from the Socioeconomic Data and Applications Center (SEDAC) via http://sedac.ciesin.columbia.edu/data/set/wildareas-v2-human-influence-index-geographic/, and had a 1‐km^2^ resolution. The proxy is based on nine variables related to human population pressure (human population density), human land use and infrastructure (built‐up areas, night‐time lights, land use/land cover), and human access (coastlines, roads, railroads, navigable rivers). Human influence may especially favour alien species through transport, use and planting of these species in the surrounding anthropogenic areas, which could increase propagule pressure in forests (i.e., spatial variation in how often species may arrive in the region, directly or indirectly due to human activity). Furthermore, human influence could have a positive effect on alien species through disturbance or nutrient deposition, which may increase the establishment success and growth of alien species in the surrounding region (and hence, facilitate spread to our forest plots) and/or in the plots.

As a measure of climatic conditions that the species experience, and to evaluate the climatic conditions associated with high species abundance, we used the standardized precipitation and evapotranspiration index (SPEI), which is a climatic drought index based on precipitation and temperature, at a spatial resolution of 0.5° (Vicente‐Serrano, Beguería & López‐Moreno, 2010). Positive values indicate humid conditions and negative values indicate dry conditions. We calculated the SPEI based on a temporal scale of 12 months, which means that the index of a specific month is based on that month and the 11 preceding months. SPEI at this time‐scale is mainly related to variations in groundwater storage (Vicente‐Serrano et al., 2010). Average monthly SPEI values were calculated based on average monthly rainfall and average monthly potential evapotranspiration. Potential evapotranspiration was derived from average monthly temperature and latitude, using the “Thornthwaite” function from the “SPEI” package in R (Begueria & Serrano, 2015). Monthly rainfall and temperature were obtained from CHELSA (climatologies at high resolution for the earth’s land surface areas), see further details in Bruelheide et al., 2019. Besides the original SPEI values, we also calculated the dissimilarity between local SPEI and species’ optimal SPEI. Optimal SPEI was calculated as the average SPEI at which the species occurred in its native range. This index indicates how close a species’ occurrence is to the centre of that species’ climatic niche, although this may not always be a good measure of “optimal” conditions (Dallas et al., 2017).

### 2.8 Statistical analyses

**Figure 2:**
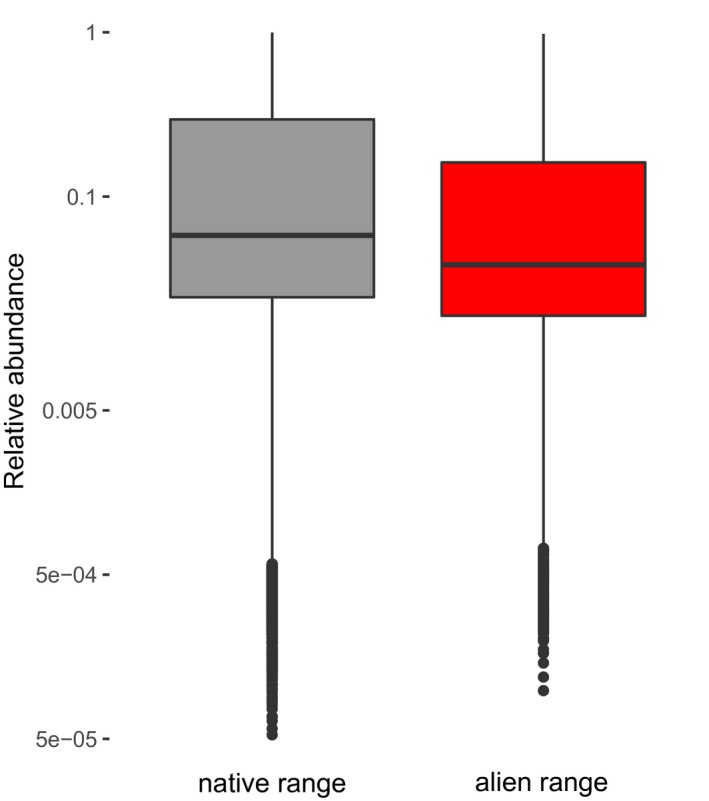
Differences in relative abundance between native (grey) and alien (red) ranges across all species. The mean relative abundance in the native range (mean = 0.21, median = 0.06) was significantly higher than the relative abundance in the alien range (mean = 0.14, median = 0.04; χ^2^ = 36.373, *p* < .001). The line in the box represents the median, and the upper and lower edges of the box represent the 95% confidence interval. For species‐specific differences in relative abundance between ranges, see Supporting Information Appendix S7 [Colour figure can be viewed at https://www.wileyonlinelibrary.com]

To test for differences in abundance between the native and alien ranges across species, we used a linear mixed‐effects model including species and plot as random intercepts, using the *lmer* function of the *lme4* package (Bates, Maechler, Bolker & Walker, 2015; Figure 2).

**Figure 3:**
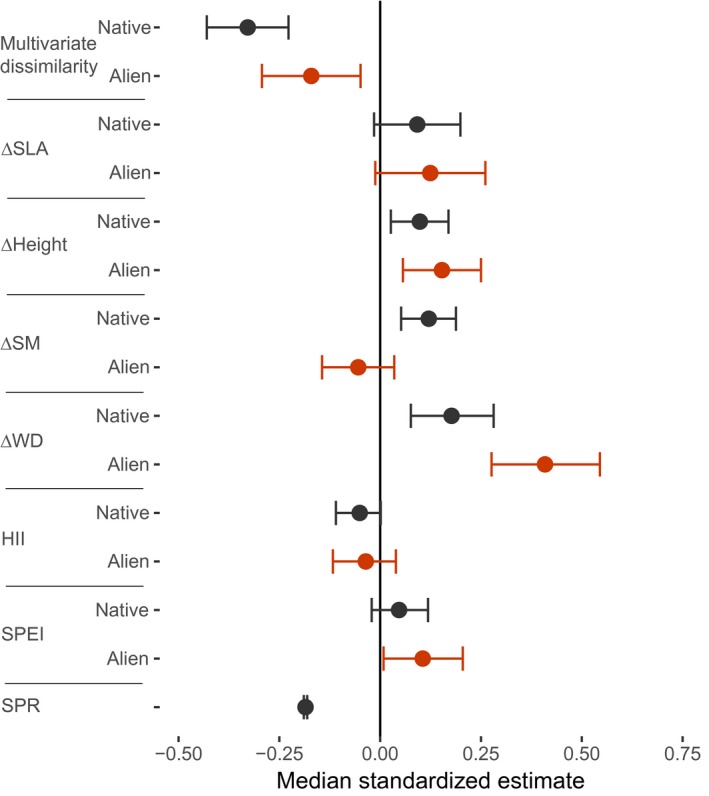
Results of the effects of trait dissimilarity, competitive trait differences and environmental conditions on local species abundance in their native (black) and alien (red) ranges. Range (native versus alien) refers to the status of the focal species in the plot. Median estimates of standardized effect sizes with 95% credible intervals for the across‐species mean effect of predictor variables on relative abundance are shown. ∆SLA = competitive differences in specific leaf area; ∆Height = competitive differences in adult height; ∆SM = competitive differences in seed mass; ∆WD = competitive differences in wood density; HII = human influence index; SPEI = standardized precipitation and evapotranspiration index; SPR = species richness. Negative effects of multivariate trait dissimilarity indicate that focal species that are functionally similar to co‐occurring species reach highest abundance. Positive and negative values for competitive trait differences (i.e., ∆SLA, ∆Height, ∆SM and ∆WD) indicate that focal species with respectively higher and lower trait values than the average co‐occurring species obtain highest abundances. For numerical values of parameter estimates, see Supporting Information Appendix S14 [Colour figure can be viewed at https://www.wileyonlinelibrary.com]

We formulated a two‐level hierarchical linear regression model (using Bayesian inference) to test for effects of multivariate trait dissimilarity, competitive trait differences [in specific leaf area (ΔSLA), plant height (ΔH), seed mass (ΔSM) and wood density (ΔWD)], human influence index (HII) and the standardized precipitation and evapotranspiration index (SPEI) on relative abundance, and to test how these effects differ between native and alien ranges and depend on species' traits. The first level of the model describes the species‐specific responses of relative abundance to the above‐mentioned predictor variables, range (native versus alien range), and the two‐way interactions of each predictor variable with range. We included a random effect of plot identity and incorporated interspecific variation in the intercept as well as in the slope parameters for all main effects. Additionally, we included woody species richness in the plot as a predictor of abundance because high richness (which is correlated with large plot size) decreases the average relative abundance of species and can therefore influence the effects of dissimilarity and competitive differences. We only included species richness to correct for this effect on relative abundance, and hence did not include an interaction between species richness and range. In the second level of the model, to better understand what drives the differences in these regression slopes among species, each regression slope was itself described by a normal linear regression sub‐model that uses species' traits (SLA, H, SM and WD) as predictors. In other words, species‐level trait values were used as predictors of the interspecific variation in regression parameters (intercept and slopes). This hierarchical model structure ensures that uncertainty in regression slopes is not neglected when estimating how they depend on species' traits (Houslay & Wilson, 2017). We did not describe interspecific variation in the interactions between range (native versus alien) and other predictors because these interaction effects on abundance (i.e., slope differences between native and alien ranges) were generally small (Figure 3). Relative abundance, the response variable, was ln‐transformed to meet assumptions of normality of the model residuals, and all variables were scaled prior to analysis by subtracting the mean and dividing by the standard deviation (Schielzeth, 2010). The variance inflation factors of the variables included in the model were all <4, indicating no problem of collinearity (Zuur, Ieno & Elphick, 2010).

To analyse the hierarchical linear regression model, we applied Bayesian inference because this can accommodate the complex hierarchical structure of our model and data. Parameter estimation was done using the jags software (Plummer, 2013) and the *rjags* package in R (Plummer, Stukalov, et al., 2016). We set uninformative priors for all parameters; for all slopes of the fixed effects and predictors of the random slopes we set priors with mean = 0 and variance = 10^3^, and for the standard deviation parameters (associated with the slopes of the fixed effects and predictors of the random slopes) we set priors with a uniform distribution between 0 and 100. Samples of the parameter posterior distribution were obtained from 3 independent Markov chains using Markov chain Monte Carlo (MCMC), each running for 10,000 iterations after a burn‐in phase of 1,000 iterations. We checked for convergence of the model visually by plotting the posterior sample distribution and trace plot of each parameter (Supporting Information Appendix S2), and by evaluating Gelman and Rubin’s univariate and multivariate potential scale reduction factors (Gelman & Rubin, 1992), using the *gelman.diag* function of the *coda* package in R (Plummer, Best, Cowles, & Vines, 2006). The univariate and multivariate potential scale reduction factors were all ≤1.01, indicating that the MCMC sampler converged. We found no significant phylogenetic signal in our model output (Supporting Information Appendix S3). A mathematical model description can be found in Supporting Information Appendix S4, and the R script in Supporting Information Appendix S5. All analyses were performed in R version 3.4.3 (R Core Team, 2017).

## 3 RESULTS

We first assessed abundance differences between native and alien ranges. We found that the relative abundance across the investigated tree species (compared to all woody species in the plot, and ranging between 0 and 1) was slightly but significantly higher in the native compared to the alien range (Figure 2), while the abundance distribution was similar between the native and alien ranges (Supporting Information Appendix S6). Results differed strongly among the species; of the 24 species classified as invasive aliens (Rejmánek & Richardson, 2013), only 6 had significantly higher abundance in the alien than in the native range, and 3 had significantly higher abundance in the native range. Of the 17 species classified as non‐invasive, only 4 showed significant differences: 2 had higher and 2 had lower abundance in the alien range (Supporting Information Appendix S7).

We then assessed which factors may determine the local‐scale abundance in the native and the alien ranges using a hierarchal linear model. The model accounted for 31% of the variation in relative abundance. We found a negative effect of multivariate trait dissimilarity in both ranges, meaning that species tend to be most abundant when they are functionally similar to their co‐occurring species (Figure 3; Supporting Information Appendix S8a). This effect was especially strong for non‐invasive alien species, and for invasive species in the native range (Supporting Information Appendix S9). We found positive effects of ∆WD and ∆H in both ranges, and of ∆SM in the native range, indicating that species with higher wood density and adult height than co‐occurring species had highest abundance in both the native and alien ranges, and species with higher seed mass than co‐occurring species had higher abundance in the native range (Figure 3, Supporting Information Appendix S8b). Abundance was negatively associated with species richness (Figure 3), positively associated with humidity (SPEI) in the alien range (only), and not associated with the HII. Results were similar when using a more balanced design with similar numbers of plots in the native and alien ranges (Supporting Information Appendix S10). SPEI similarity to species' optimal conditions was not associated with abundance (Supporting Information Appendix S11).

**Figure 4:**
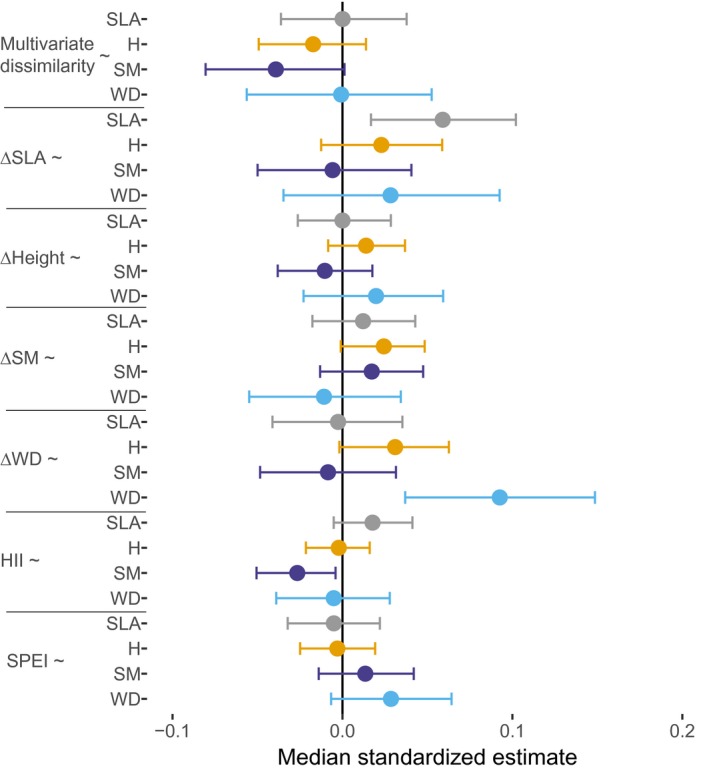
The effect of traits (grey: specific leaf area (SLA); orange: adult height (H); purple: seed mass (SM); blue: wood density (WD)) on species‐specific differences in the slopes of the relationships of predictors (multivariate trait dissimilarity, competitive differences in each of the four traits (ΔSLA, ΔH, ΔSM, ΔWD), human influence index (HII) and standardized precipitation and evapotranspiration index (SPEI)) with relative abundance. The median estimate of standardized effect size with 95% credible intervals is given. For meaning of abbreviations, see Figure 3 legend. Positive effects (e.g., “∆SLA~SLA”) indicate that the trait (SLA) positively affects the slope of the relationship between the predictor (∆SLA) and abundance. Specifically, the effect of the predictor (∆SLA) on abundance is more positive for species with high values of that trait (SLA). For numerical values of parameter estimates, see Supporting Information Appendix S14 [Colour figure can be viewed at https://www.wileyonlinelibrary.com]

Species‐specific SLA values increased the effect of ∆SLA on abundance, indicating that species with high SLA experienced a stronger effect of ∆SLA on their abundance than species with low SLA (Figure 4). Similarly, species‐specific WD increased the effect of ∆WD on abundance. These results were robust to excluding gymnosperms, papaya (*Carica papaya*) and a palm species (*Syagrus romanzoffiana*) (Supporting Information Appendix S12), but effects on abundance of gymnosperms themselves were weak (Supporting Information Appendix S13). Thus, the stronger effects of ∆SLA and ∆WD for species with high SLA and WD mainly applied to angiosperm trees.

## 4 DISCUSSION

We evaluated differences in local abundance of tree species between their native and alien ranges, and the factors accounting for abundance in both ranges. We expected that alien species, and in particular invasive alien species, would be more abundant in the alien compared to the native range. However, we found that species tend to be slightly less abundant in their alien range (Figure 2). These results indicate that, even though species are classified as invasive, their local‐scale abundance varies strongly. A lower abundance in the alien range has also been found for herbaceous species (Firn et al., 2011), and could be explained by various factors, such as local‐scale competition by resident tree species, less favourable environmental conditions in the alien range, and their time since arrival. It could also be that abundance in the alien range is not yet in steady state and is still increasing due to ongoing invasion.

We expected that multivariate trait dissimilarity to co‐occurring trees would enhance species abundance through niche complementarity (hypothesis 1). In contrast, we found a negative relationship between multivariate trait dissimilarity and abundance in both the native and alien ranges (Figure 3), suggesting an over‐riding influence of environmental–biotic filtering. Furthermore, we expected that species with higher values for competitive traits than co‐occurring trees would be more abundant (hypothesis 2). Indeed, we found that species that are taller and have denser wood than their neighbours tended to be more abundant in both their native and alien ranges, and species with larger seeds were more abundant in the native range (Figure 3, Supporting Information Appendix S8b). Hence, high multivariate similarity, but competitive differences in specific traits, lead to highest abundance.

### 4.1 Environmental–biotic filtering drives abundance in native and alien ranges

Our finding that tree species with low trait dissimilarity to co‐occurring trees are relatively more abundant is consistent with a recent global study that showed weak effects of trait dissimilarity on tree growth (Kunstler et al., 2016). However, our results sharply contrast with hypothesis 1, and with results of many studies in the invasion literature on different vegetation types, which find that local‐scale species abundance is mainly determined by biotic interactions and niche differentiation (Carboni et al., 2013). Trees in forest ecosystems may experience strong environmental–biotic filtering at local spatial scales (e.g., plots < 1 ha) because the dense vegetation causes strong light limitation, which could explain our observed negative effect of trait dissimilarity on abundance (Montesinos‐Navarro et al., 2018). Thus, our results contradict the idea that functionally dissimilar alien species would experience reduced competition with native species, and would become most dominant (Rundel, Dickie & Richardson, 2014). Functional dissimilarity may be important in more open ecosystems such as grasslands (Carboni et al., 2016), but our results, and the results of Kunstler et al. (2016), suggest that this is not the case for tree species in forest ecosystems.

### 4.2 Higher wood density, height and seed mass than co‐occurring trees increase abundance in native and alien ranges

We expected, and found, that species that grow taller and have denser wood and larger seeds compared to their neighbours reach highest abundance (Figure 3, Supporting Information Appendix S8b). Species with taller adult stature than co‐occurring species (i.e., positive ∆H) can access higher light conditions that allow fast growth, and contribute disproportionally to woody abundance in the plot (Slik et al., 2013), which may enhance their abundance levels. WD is a generally important trait for trees in forests, as high WD is related to shade tolerance (Ameztegui et al., 2017; Markesteijn et al., 2011). Although alien species with “acquisitive” trait values (e.g., species with low WD that grow faster in diameter and height) are usually most successful in open and disturbed ecosystems (Kleunen, Weber, et al., 2010; Tecco, Díaz, Cabido & Urcelay, 2010), more conservative trait values (e.g., higher shade tolerance and WD) may be of general importance in forests (Martin et al., 2009; van der Sande et al., 2017). The effect of ∆WD, however, tended to be stronger in the alien compared to the native range (Figure 3), suggesting a higher importance of shade tolerance, possibly because light limitation suppresses tree abundance more strongly in the alien range. The underlying reason for this may be that trees are typically younger and smaller in the alien range, and therefore suffer more light limitation than in the native range. Further research needs to demonstrate whether and why light limitation is a stronger constraint in the alien compared to the native range.

The positive effect of ∆SM in the native range is consistent with the idea that light limitation creates a strong filter, as larger seeds are associated with enhanced recruitment success in the forest understorey (Dalling & Hubbell, 2002). ∆SM did not, however, affect abundance in the alien range. There is a known trade‐off between seed number and seed size, and this could suggest that seed number is more important to achieving higher abundance in the alien range than it is in the native range. This novel finding could indicate that propagule pressure not only initiates, but also sustains, invasion. Small seeds enhance invasion in open ecosystems (Taylor et al., 2016), but could also enhance abundance in forests if dispersal is a limiting factor. The balance between the importance of seed number and seed size may also depend on the time since establishment in the alien range. With longer time since arrival to a new area, abundance may be less limited by the lack of arriving seeds (i.e., seed number) and relatively more by establishment of seedlings (i.e., seed size).

### 4.3 Human influence is not associated with local tree abundance

We expected that tree abundance in the alien range would be highest with strong human influence in the region, as human influence may increase propagule pressure (Gallardo et al., 2015) through higher planting intensity of alien species in the surrounding region, which then disperse their seeds to nearby forests, and/or through higher disturbance and human‐facilitated seed dispersal (intentional or unintentional) (Dainese et al., 2017; Hulme, 2014). We found, however, that the relationship between abundance of alien species and human influence was not significant (Figure 3). This indicates that, although propagule pressure is known to be important for enhancing regional abundance and invasiveness of woody alien species (Bucharova & van Kleunen, 2009; Feng & van Kleunen, 2016), and for local‐scale abundance of life forms other than trees (Chytrý et al., 2008), human influence (at least as quantified in this study) may not predict local‐scale abundance of alien tree species in forests. The lack of effect of human influence on tree abundance could be caused by a weaker relationship between human influence and propagule pressure for trees than for other life forms, because many alien tree species were first introduced in plantations that were far from built‐up areas and night‐time lights (which are key components of the HII). Our measure of human influence might not capture the presence of nearby plantations (and hence, seed source areas), or key aspects of human influence on secondary forests. Proximity of plantations could be a predictor of invasive alien tree species abundance, explaining the weaker role of environmental–biotic filtering and height differences in accounting for their abundance (Supporting Information Appendix S9a).

### 4.4 Species‐specific differences in mechanisms underlying abundance

We expected that species may vary in the importance of environmental–biotic filtering, niche differentiation effects and competitive trait differences for their local abundance. Specifically, abundance of species with a conservative growth strategy (e.g., with high WD and low SLA) may be most strongly affected by environmental–biotic filtering, because strong filtering limits the abundance of other, potentially more competitive species. The abundance of acquisitive species (e.g., with low WD and high SLA), however, may mostly depend on competitive trait differences, which can confer an advantage over slower growing conservative species in high‐light environments. The positive effect of SLA on the relationship between ∆SLA and abundance indicates that especially acquisitive species may indeed gain advantage from competitive trait differences (even though, on average across species, ∆SLA did not affect abundance, Figure 3). The positive effect of WD on the relationship between ∆WD and abundance indicates that especially conservative species with dense wood may gain an advantage. These results may indicate that two different strategies—being more acquisitive and being more durable than co‐occurring species—can both provide competitive advantage and increase abundance (Guo et al., 2018). Alternatively, these results, and the absence of effects of SLA and WD on other trait effects (e.g., multivariate dissimilarity, ∆H, ∆SM) on abundance, may indicate that species with quite extreme values of SLA or WD may gain the strongest advantage of ∆SLA and ∆WD on abundance.

The negative effect of seed mass on the relationship between HII and abundance (Figure 4) indicates that species with small seeds experience more positive effects of human influence, possibly because smaller seeded species tend to be better adapted to human disturbances.

## 5 CONCLUSIONS AND APPLICATIONS

We show that tree species have similar levels of abundance in their native and alien ranges, and show striking similarities in the factors that are associated with abundance in both ranges. This contradicts hypotheses and results for other life forms in the invasion literature, which suggest that alien species are most successful when their niches differ from those of the resident species or they have novel traits. Instead, our results indicate that tree species that are broadly functionally similar to co‐occurring native trees, but have higher values for traits linked with competitiveness, become most abundant in both the native and alien ranges. These effects were especially strong for species with high wood density, suggesting a general importance of light limitation as a strong filter in forest ecosystems. These results are consistent with the notion that similar ecological mechanisms apply to native and alien species (Oduor, Leimu, Kleunen & Mack, 2016), and suggest that we can use information on species from their native range to predict how locally dominant they may become elsewhere.

## AUTHOR CONTRIBUTIONS

MTvdS and TMK conceived the idea of the study; HB, WD, JD, FE, SH, MvK, HK, J.Pergl, OP, P.Pyšek, PW, MW, FJ, UJ, JK and BJA were involved in establishing the GloNAF, sPlot and TRY databases and helped develop the idea of the study; FA, IA, EB, MC, MD, MDS, JF, RF, VG, GRG, AGG, BJA, UJ, EK, SK, KK, MM, ÜN, RKP, JP, MS, MS, P.Petřík, PBR, BS, MS, CV, TJSW and TW provided data; MTvdS analysed the data and J.Pagel assisted with data analyses; MTvdS and TMK interpreted the results; MTvdS wrote the main draft of the manuscript; all authors discussed the results and revised the manuscript.

## BIOSKETCH

This research project was carried out as a collaborative effort among three large platforms (GloNAF, sPlot and TRY), and among people interested in understanding the functioning of alien tree species at global scale. We think that such a large‐scale comparative approach enables the generation of insights and generalizations regarding the functioning of alien trees.

## Data Availability

The data used in this manuscript are published in the data repository of the German Centre for Integrative Biodiversity Research (iDiv): https://doi.org/10.25829/idiv.1805-12-2697.
