# Supplementary material for: Similar factors underlie tree abundance in forests in native and alien ranges
Source: Glob Ecol Biogeogr. 2019 Dec 1;29(2):281–94. doi: 10.1111/geb.13027 (PMC7006795; doi:10.1111/geb.13027)
Supplement: Supplementary file 1 [file GEB-29-281-s001.docx]

**Appendix S1:** Species-specific information. OC=Oceania; NA=North America; EU=Europe; AS=Asia; SA=South America; AF=Africa. Alien continents refer to those from where data were included, and do not necessarily cover all alien continents. Species with an asterisk (*) are invasive alien species.

| **Species** | **Family** | **SLA**  **(cm^2^ g^-1^)** | **Seed mass (mg)** | **Plant height (m)** | **Stem density**  **(g cm^-3^)** | **Continent of origin** | **Alien continent(s)** | **Sample size native range** | **Sample size alien range** |
| --- | --- | --- | --- | --- | --- | --- | --- | --- | --- |
| *Acacia mearnsii** | Leguminosae | 69.48 | 13.71 | 8.10 | 0.64 | OC | EU | 50 | 6 |
| *Acer negundo** | Sapindaceae | 207.19 | 33.59 | 13.48 | 0.50 | NA | EU | 742 | 658 |
| *Acer platanoides** | Sapindaceae | 211.52 | 95.58 | 21.22 | 0.57 | EU | NA, SA | 16239 | 52 |
| *Acer pseudoplatanus* | Sapindaceae | 147.63 | 86.89 | 28.00 | 0.52 | EU | NA, SA, AF | 46278 | 100 |
| *Acer saccharinum** | Sapindaceae | 205.10 | 126.23 | 31.05 | 0.68 | NA | AS, EU | 206 | 13 |
| *Ailanthus altissima** | Simaroubaceae | 146.46 | 29.46 | 19.93 | 0.51 | AS | EU, NA, OC | 4 | 431 |
| *Albizia julibrissin** | Leguminosae | 201.90 | 38.36 | 10.83 | 0.62 | AS | NA | 139 | 81 |
| *Carica papaya* | Caricaceae | 436.25 | 20.13 | 4.03 | 0.25 | SA | AS, OC | 5 | 3 |
| *Carpinus betulus* | Betulaceae | 232.13 | 43.91 | 18.82 | 0.67 | EU, AS | NA, SA | 38880 | 12 |
| *Celtis sinensis** | Cannabaceae | 183.39 | 91.84 | 17.13 | 0.58 | AS | OC | 164 | 7 |
| *Cordia myxa* | Boraginaceae | 136.84 | 273.35 | 7.23 | 0.38 | AS | AF, EU | 4 | 4 |
| *Dacryodes edulis* | Burseraceae | 114.63 | 2970.73 | 24.10 | 0.52 | AF | NA, SA | 6 | 13 |
| *Fagus sylvatica* | Fagaceae | 185.83 | 234.40 | 27.43 | 0.69 | EU | SA, NA | 69916 | 42 |
| *Ficus microcarpa** | Moraceae | 88.32 | 0.39 | 10.01 | 0.48 | AS | SA, NA, AF | 185 | 17 |
| *Fraxinus americana** | Oleaceae | 294.88 | 39.91 | 8.20 | 0.63 | NA | SA, EU, | 3330 | 52 |
| *Gleditsia triacanthos** | Leguminosae | 231.41 | 159.05 | 26.52 | 0.68 | NA | SA, EU, OC | 122 | 44 |
| *Hovenia dulcis** | Rhamnaceae | 282.22 | 27.24 | 17.45 | 0.55 | AS | SA | 7 | 119 |
| *Juglans nigra* | Juglandaceae | 215.05 | 6591.25 | 30.94 | 0.54 | NA | EU, SA | 633 | 68 |
| *Larix kaempferi* | Pinaceae | 149.00 | 5.75 | 28.76 | 0.47 | AS | EU | 27 | 498 |
| *Magnolia grandiflora* | Magnoliaceae | 88.80 | 67.30 | 21.91 | 0.45 | NA | SA | 109 | 4 |
| *Morus alba** | Moraceae | 170.95 | 4.47 | 7.12 | 0.62 | AS | EU, SA, NA, OC | 8 | 109 |
| *Pinus banksiana** | Pinaceae | 50.96 | 3.60 | 5.23 | 0.48 | NA | EU | 236 | 10 |
| *Pinus caribaea** | Pinaceae | 52.27 | 17.04 | 42.60 | 0.53 | NA | OC | 3 | 3 |
| *Pinus elliottii** | Pinaceae | 40.39 | 30.13 | 19.12 | 0.64 | NA | OC | 194 | 4 |
| *Pinus strobus** | Pinaceae | 66.14 | 17.82 | 42.43 | 0.38 | NA | EU | 2438 | 470 |
| *Piper aduncum** | Piperaceae | 221.63 | 1.16 | 9.90 | 0.44 | NA, SA | AS | 202 | 3 |
| *Platanus occidentalis* | Platanaceae | 190.68 | 3.06 | 34.35 | 0.46 | NA | EU | 655 | 16 |
| *Populus deltoides** | Salicaceae | 116.61 | 1.28 | 28.14 | 0.44 | NA | EU, OC | 79 | 1981 |
| *Prunus avium* | Rosaceae | 158.72 | 181.25 | 15.62 | 0.60 | EU | NA | 21771 | 95 |
| *Pseudotsuga menziesii** | Pinaceae | 73.51 | 11.24 | 56.35 | 0.45 | NA | EU | 1046 | 1269 |
| *Psidium guajava** | Myrtaceae | 109.17 | 10.64 | 3.11 | 0.69 | NA, SA | AS, OC, AF | 17 | 5 |
| *Pyrus calleryana** | Rosaceae | 130.64 | 5.92 | 6.98 | 0.60 | AS | NA | 3 | 3 |
| *Quercus palustris* | Fagaceae | 91.78 | 938.22 | 27.81 | 0.65 | NA | EU, AF | 204 | 56 |
| *Quercus rubra** | Fagaceae | 157.31 | 1636.71 | 25.78 | 0.66 | NA | EU, AF | 5302 | 5714 |
| *Robinia pseudoacacia** | Leguminosae | 182.97 | 23.99 | 19.03 | 0.68 | NA | EU, AS, OC, AF | 1898 | 3327 |
| *Salix fragilis** | Salicaceae | 110.37 | 0.10 | 10.30 | 0.41 | EU | NA | 1246 | 3 |
| *Schinus terebinthifolia* | Anacardiaceae | 102.41 | 19.42 | 5.61 | 0.65 | SA | OC | 84 | 176 |
| *Spondias mombin* | Anacardiaceae | 140.34 | 1294.24 | 21.35 | 0.38 | SA | AF, AS | 125 | 85 |
| *Syagrus romanzoffiana* | Arecaceae | 120.57 | 2778.65 | 1.38 | 0.46 | SA | OC | 421 | 14 |
| *Theobroma cacao* | Malvaceae | 186.41 | 1312.59 | 11.60 | 0.43 | SA | AS, AF | 83 | 13 |
| *Thuja occidentalis* | Cupressaceae | 47.36 | 1.75 | 18.35 | 0.35 | NA | EU | 298 | 4 |

**Appendix S2:** Trace plots (left column) and posterior sample distributions (right column) of each explanatory variable. The red, green and black lines in the trace plots indicate the three chains.
