# Supplementary material for: Similar factors underlie tree abundance in forests in native and alien ranges
Source: Glob Ecol Biogeogr. 2019 Dec 1;29(2):281–94. doi: 10.1111/geb.13027 (PMC7006795; doi:10.1111/geb.13027)
Supplement: Supplementary file 2 [file GEB-29-281-s002.pdf]

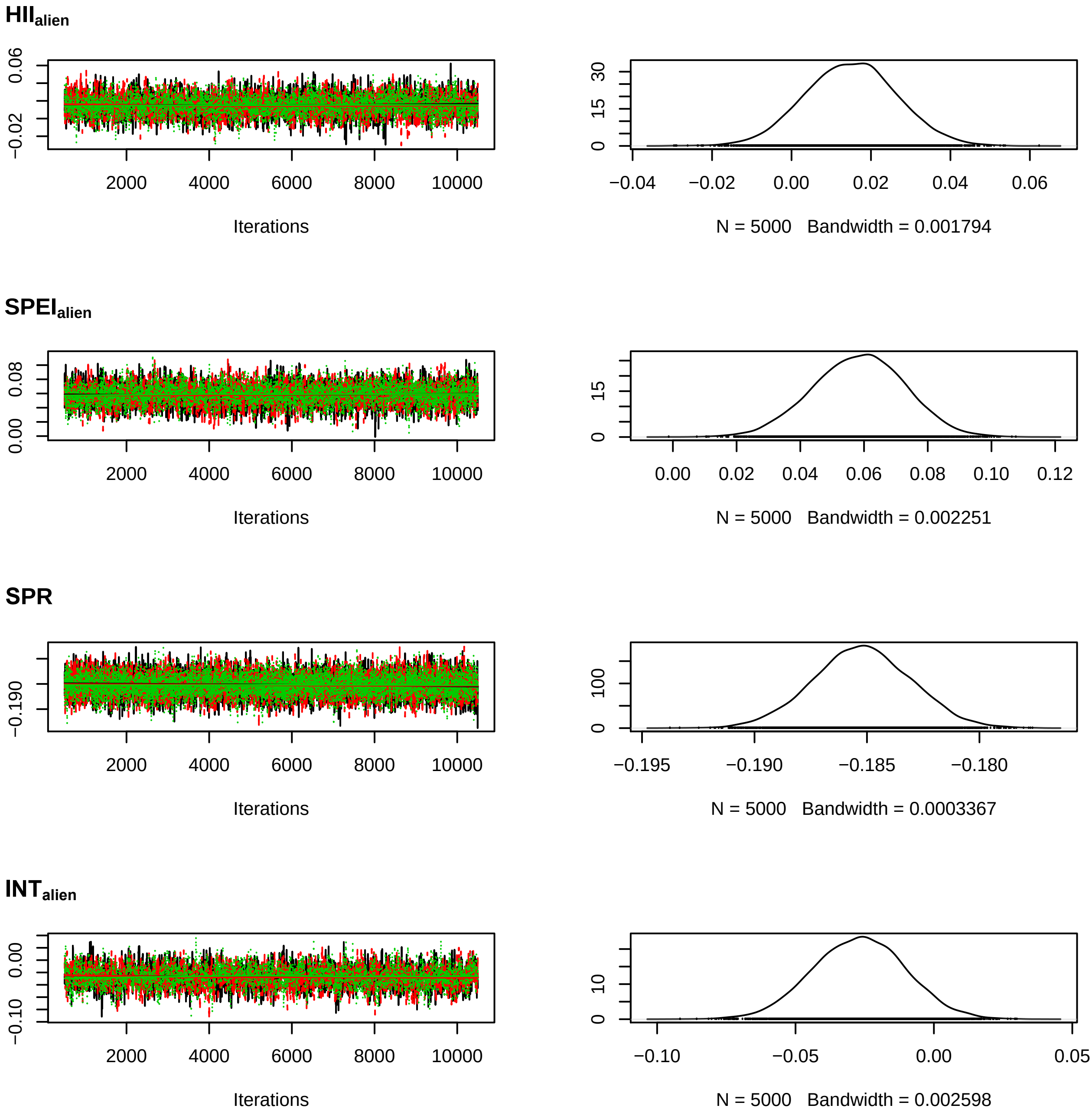
HII alien
SPEI alien
SPR
INT alien
Iterations
2000
4000
6000
8000
10000
N = 5000 Bandwidth = 0.001794
N = 5000 Bandwidth = 0.002251
N = 5000 Bandwidth = 0.0003367
N = 5000 Bandwidth = 0.002598
−0.04
−0.02
0.00
0.02
0.04
0.06
0.08
0.10
0.12
−0.195
−0.190
−0.185
−0.180
−0.10
−0.05
0.05
0
10
15
30
100

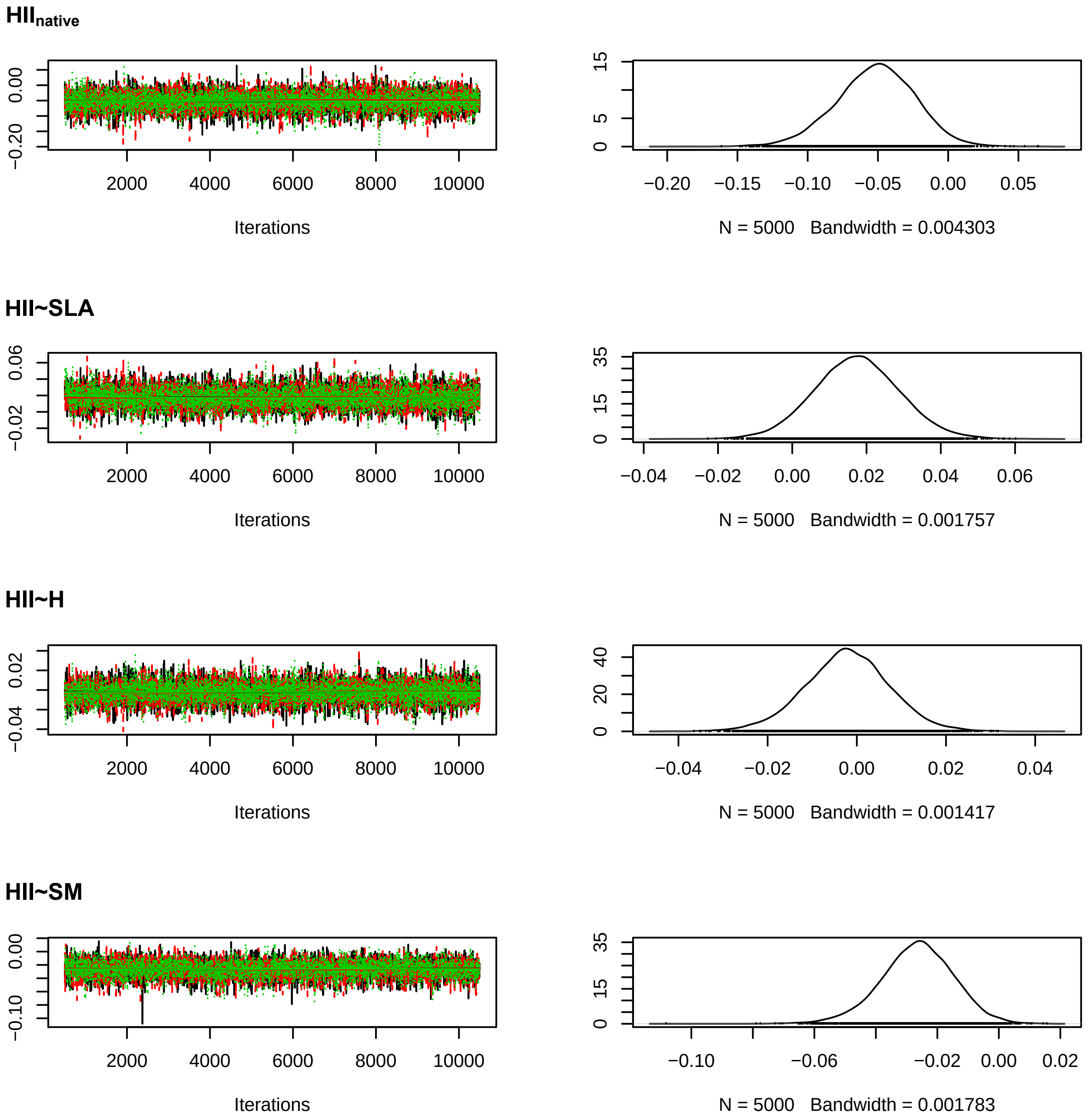

HIInative
0.00
−0.20
2000
4000
6000
8000
10000
Iterations
15
5
0
−0.20
−0.15
−0.10
−0.05
0.00
0.05
N = 5000 Bandwidth = 0.004303
HII~SLA
0.06
−0.02
2000
4000
6000
8000
10000
Iterations
35
15
0
−0.04
−0.02
0.00
0.02
0.04
0.06
N = 5000 Bandwidth = 0.001757
HII~H
0.02
−0.04
2000
4000
6000
8000
10000
Iterations
40
20
0
−0.04
−0.02
0.00
0.02
0.04
N = 5000 Bandwidth = 0.001417
HII~SM
0.00
−0.10
2000
4000
6000
8000
10000
Iterations
35
15
0
−0.10
−0.06
−0.02
0.00
0.02
N = 5000 Bandwidth = 0.001783

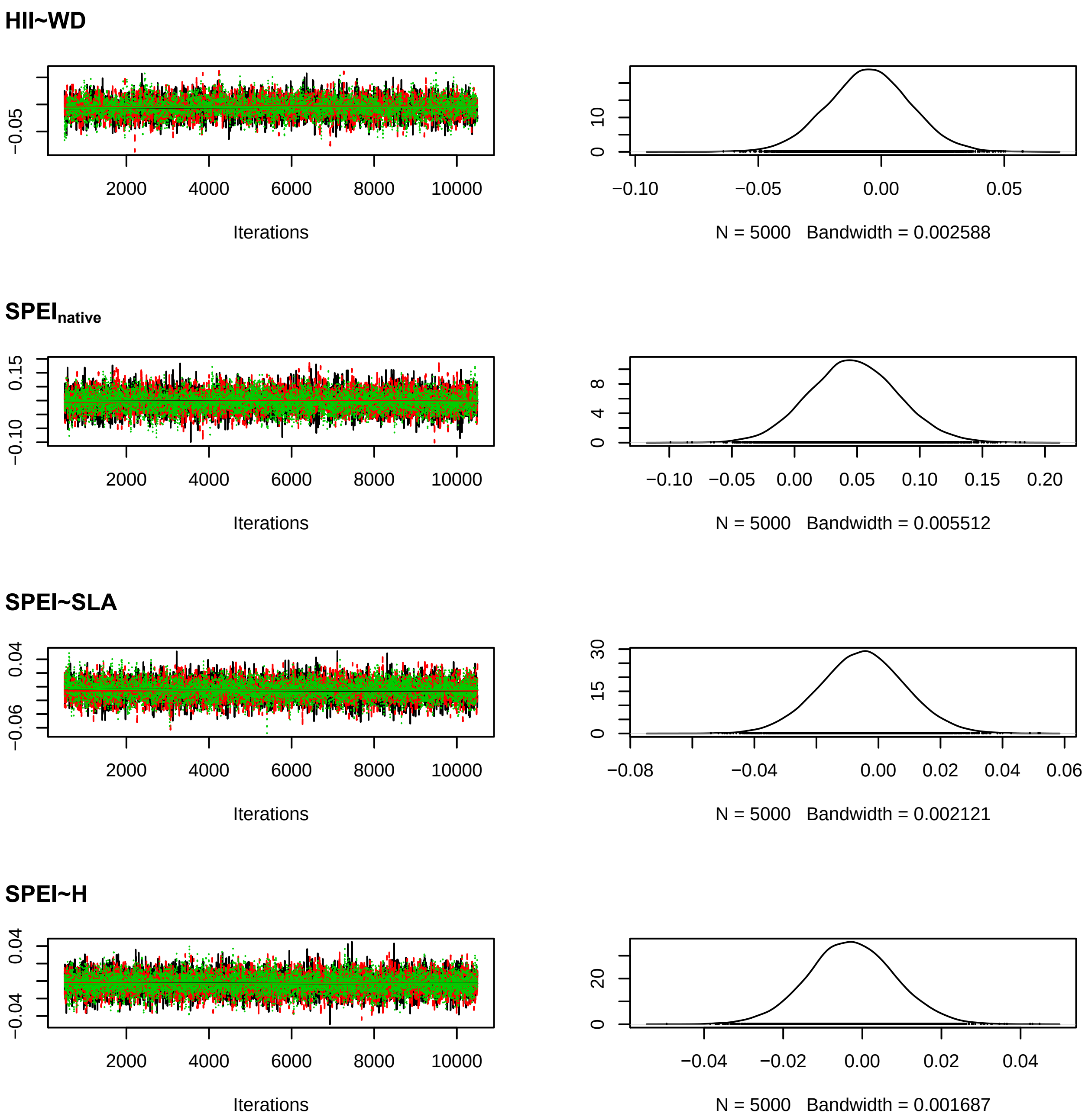
HII~WD
Iterations
N = 5000 Bandwidth = 0.002588
$SPEI_{native}$
Iterations
N = 5000 Bandwidth = 0.005512
SPEI~SLA
Iterations
N = 5000 Bandwidth = 0.002121
SPEI~H
Iterations
N = 5000 Bandwidth = 0.001687

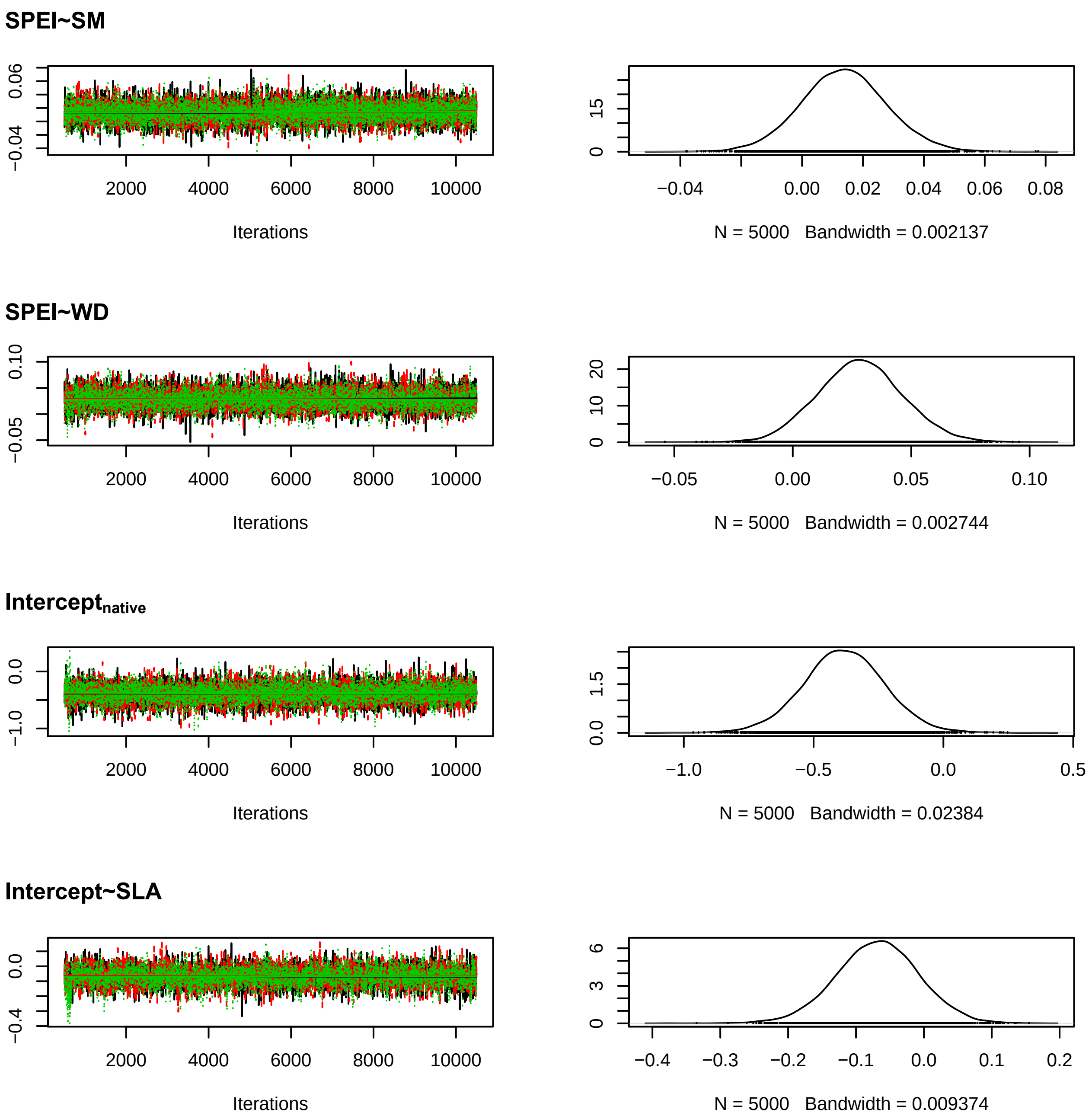
SPEI~SM
Iterations
N = 5000 Bandwidth = 0.002137
SPEI~WD
Iterations
N = 5000 Bandwidth = 0.002744
Intercept$_{native}$
Iterations
N = 5000 Bandwidth = 0.02384
Intercept~SLA
Iterations
N = 5000 Bandwidth = 0.009374

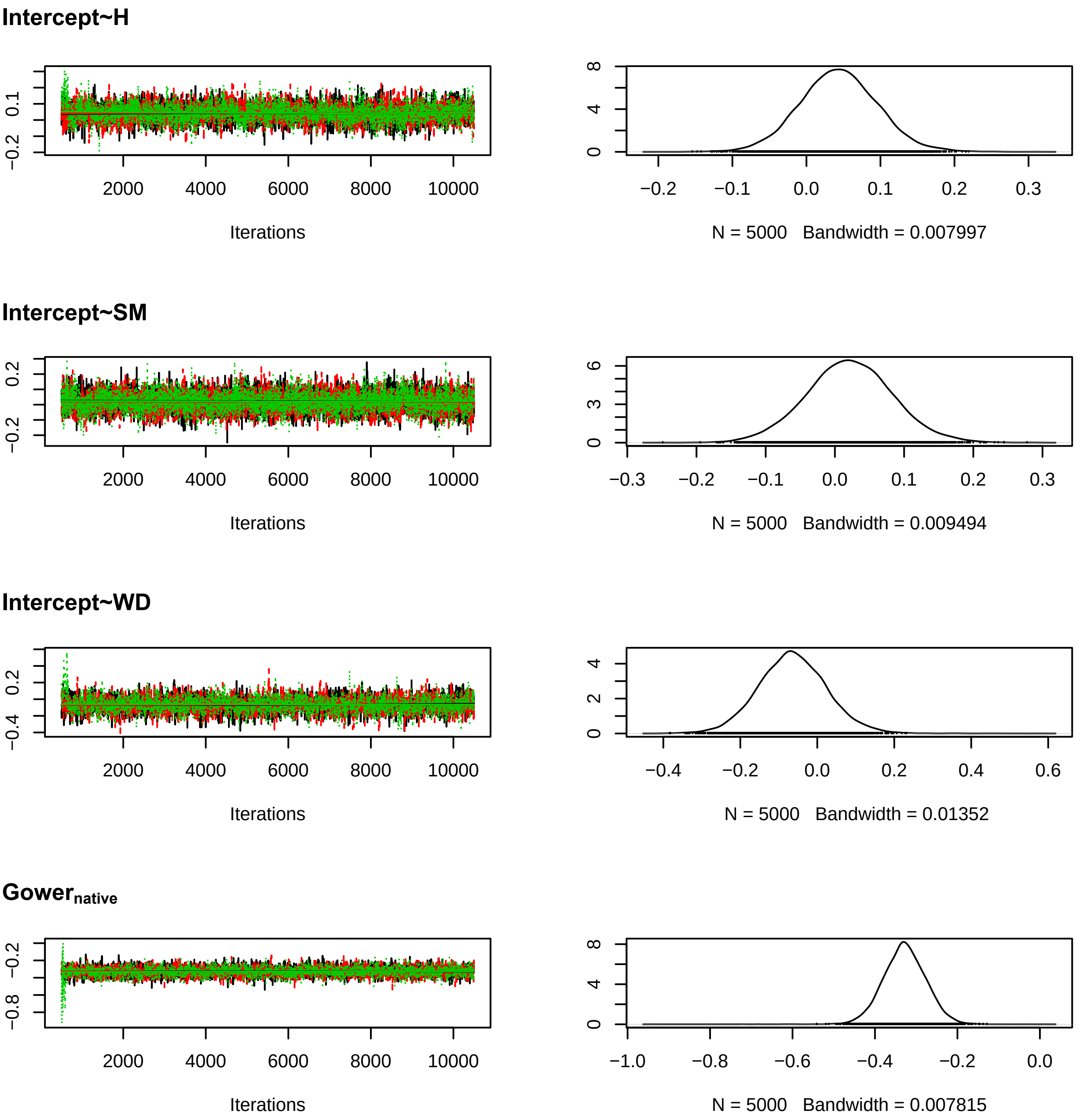

Intercept~H
0.1
−0.2
2000
4000
6000
8000
10000
Iterations
8
4
0
−0.2
−0.1
0.0
0.1
0.2
0.3
N = 5000   Bandwidth = 0.007997
Intercept~SM
0.2
−0.2
2000
4000
6000
8000
10000
Iterations
6
3
0
−0.3
−0.2
−0.1
0.0
0.1
0.2
0.3
N = 5000   Bandwidth = 0.009494
Intercept~WD
0.2
−0.4
2000
4000
6000
8000
10000
Iterations
4
2
0
−0.4
−0.2
0.0
0.2
0.4
0.6
N = 5000   Bandwidth = 0.01352
Gower$_{native}$
−0.2
−0.8
2000
4000
6000
8000
10000
Iterations
8
4
0
−1.0
−0.8
−0.6
−0.4
−0.2
0.0
N = 5000   Bandwidth = 0.007815

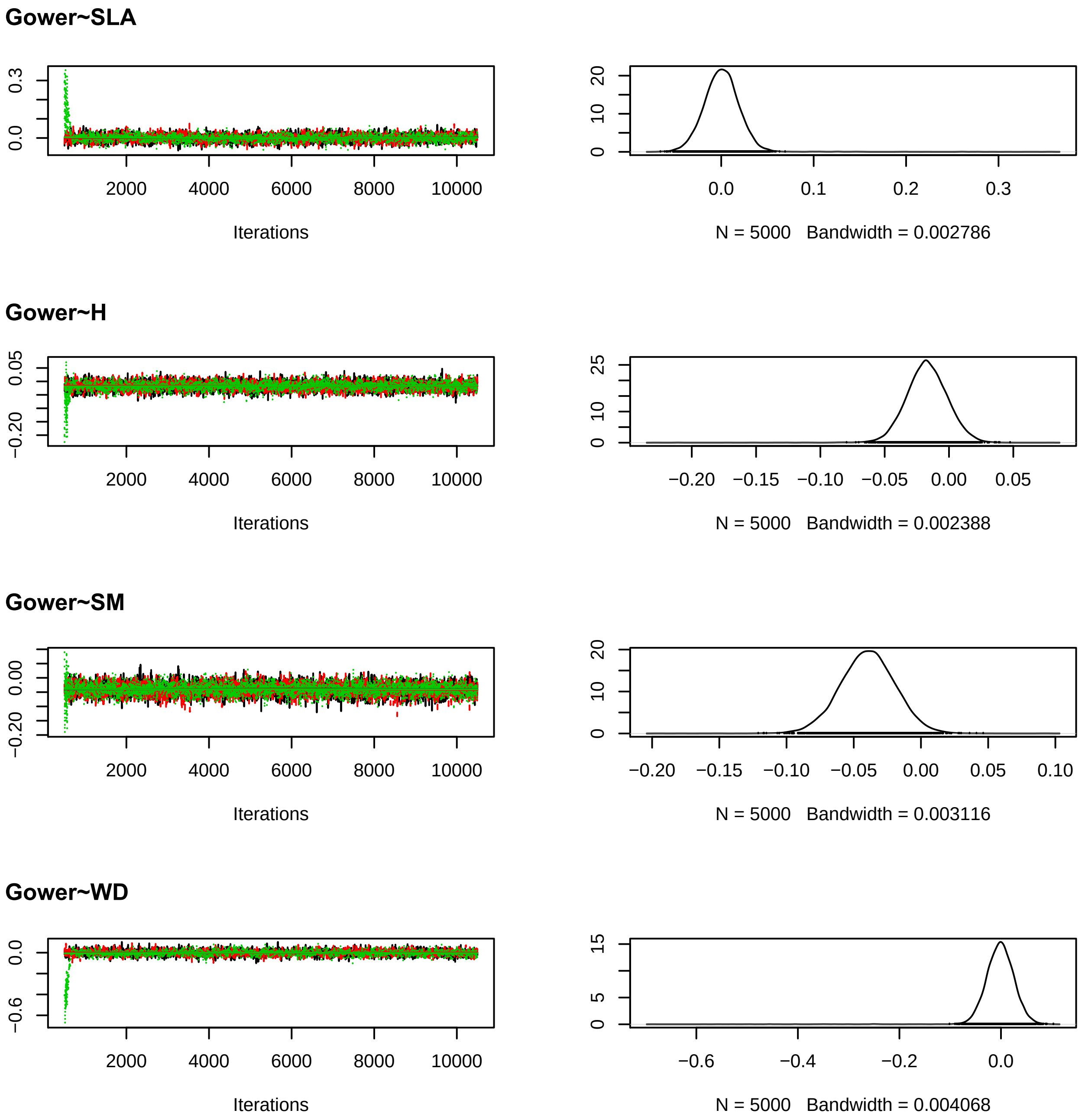

Gower~SLA
0.0
0.3
2000
4000
6000
8000
10000
Iterations
0
10
20
0.0
0.1
0.2
0.3
N = 5000 Bandwidth = 0.002786
Gower~H
−0.20
0.05
2000
4000
6000
8000
10000
Iterations
0
10
25
−0.20
−0.15
−0.10
−0.05
0.00
0.05
N = 5000 Bandwidth = 0.002388
Gower~SM
−0.20
0.00
2000
4000
6000
8000
10000
Iterations
0
10
20
−0.20
−0.15
−0.10
−0.05
0.00
0.05
0.10
N = 5000 Bandwidth = 0.003116
Gower~WD
−0.6
0.0
2000
4000
6000
8000
10000
Iterations
0
5
15
−0.6
−0.4
−0.2
0.0
N = 5000 Bandwidth = 0.004068

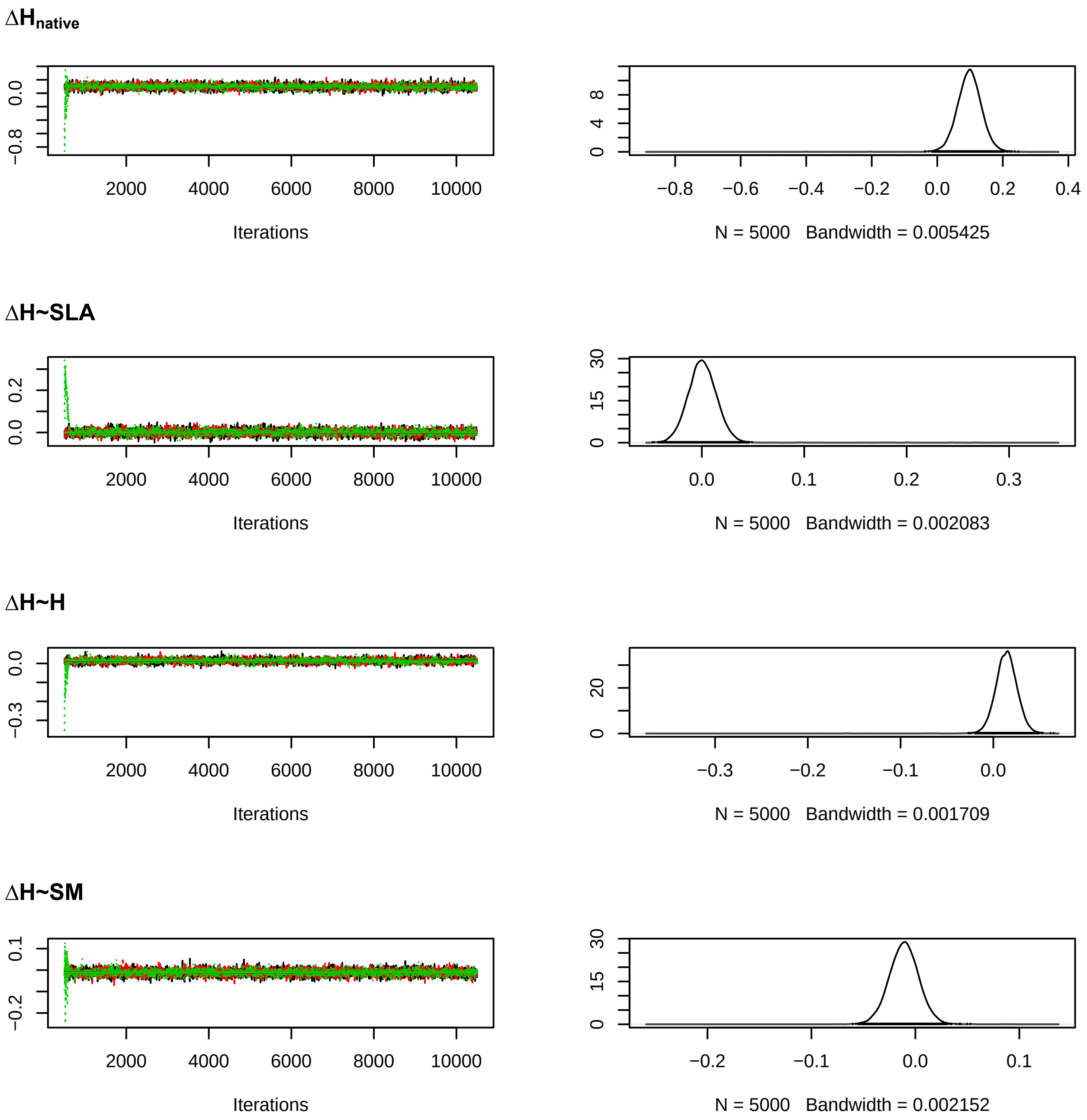

ΔH$_{native}$
Iterations
N = 5000 Bandwidth = 0.005425
ΔH~SLA
Iterations
N = 5000 Bandwidth = 0.002083
ΔH~H
Iterations
N = 5000 Bandwidth = 0.001709
ΔH~SM
Iterations
N = 5000 Bandwidth = 0.002152

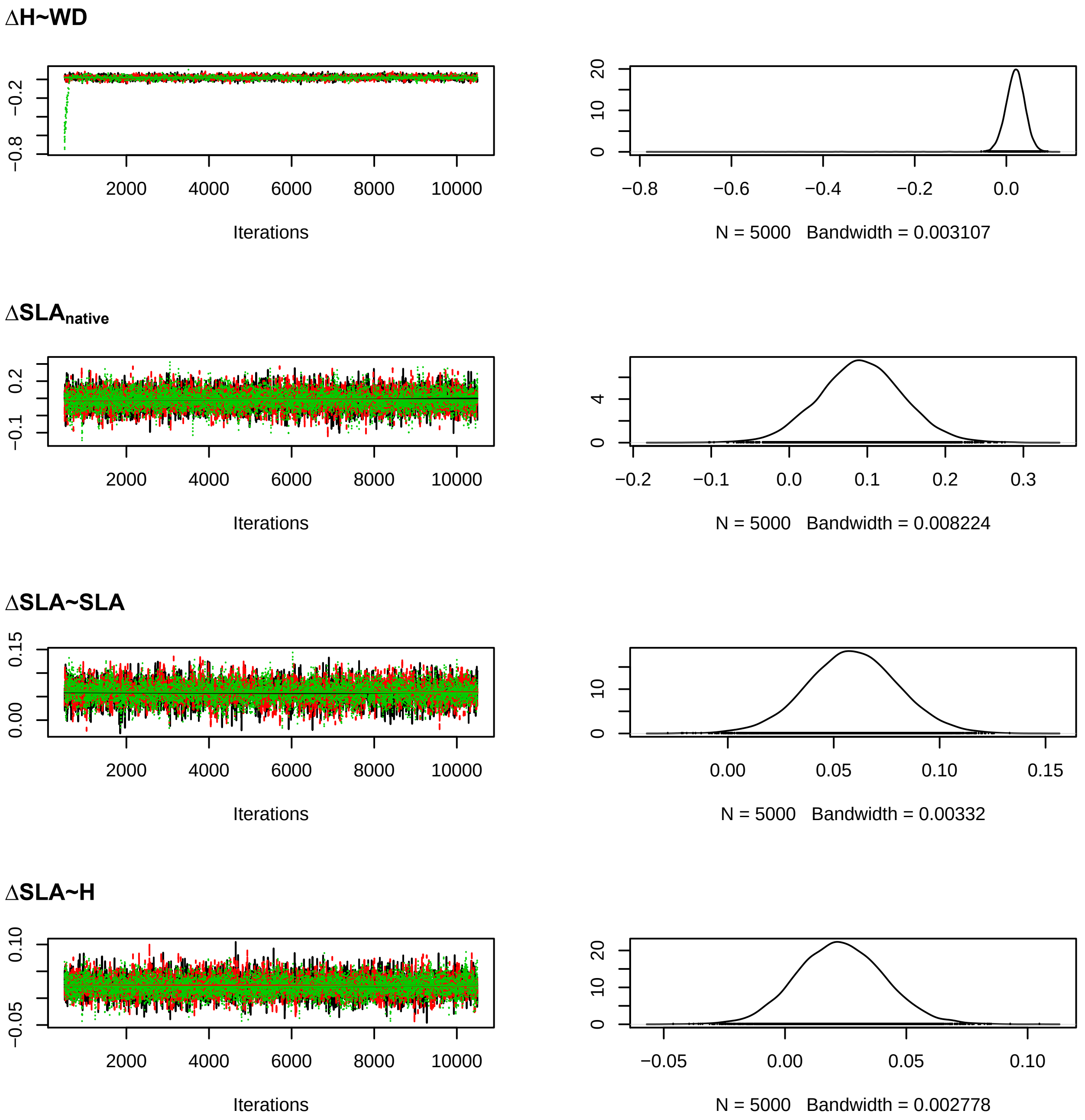

ΔH~WD
−0.8
−0.2
2000
4000
6000
8000
10000
Iterations
0
10
20
−0.8
−0.6
−0.4
−0.2
0.0
N = 5000 Bandwidth = 0.003107
ΔSLA$_{native}$
−0.1
0.2
2000
4000
6000
8000
10000
Iterations
0
4
−0.2
−0.1
0.0
0.1
0.2
0.3
N = 5000 Bandwidth = 0.008224
ΔSLA~SLA
0.00
0.15
2000
4000
6000
8000
10000
Iterations
0
10
0.00
0.05
0.10
0.15
N = 5000 Bandwidth = 0.00332
ΔSLA~H
−0.05
0.10
2000
4000
6000
8000
10000
Iterations
0
10
20
−0.05
0.00
0.05
0.10
N = 5000 Bandwidth = 0.002778

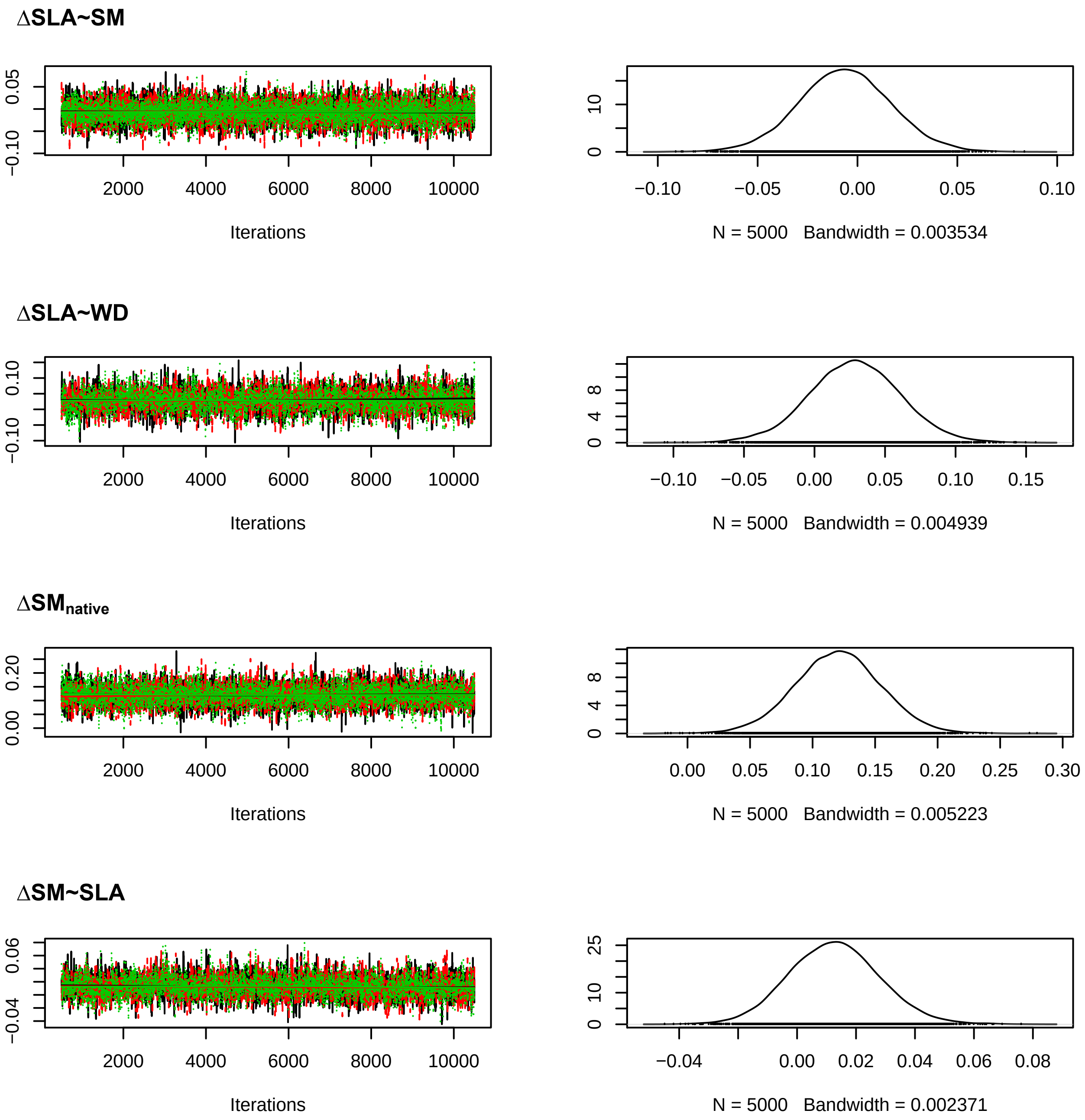

ΔSLA~SM
Iterations
N = 5000 Bandwidth = 0.003534
ΔSLA~WD
Iterations
N = 5000 Bandwidth = 0.004939
ΔSM native
Iterations
N = 5000 Bandwidth = 0.005223
ΔSM~SLA
Iterations
N = 5000 Bandwidth = 0.002371

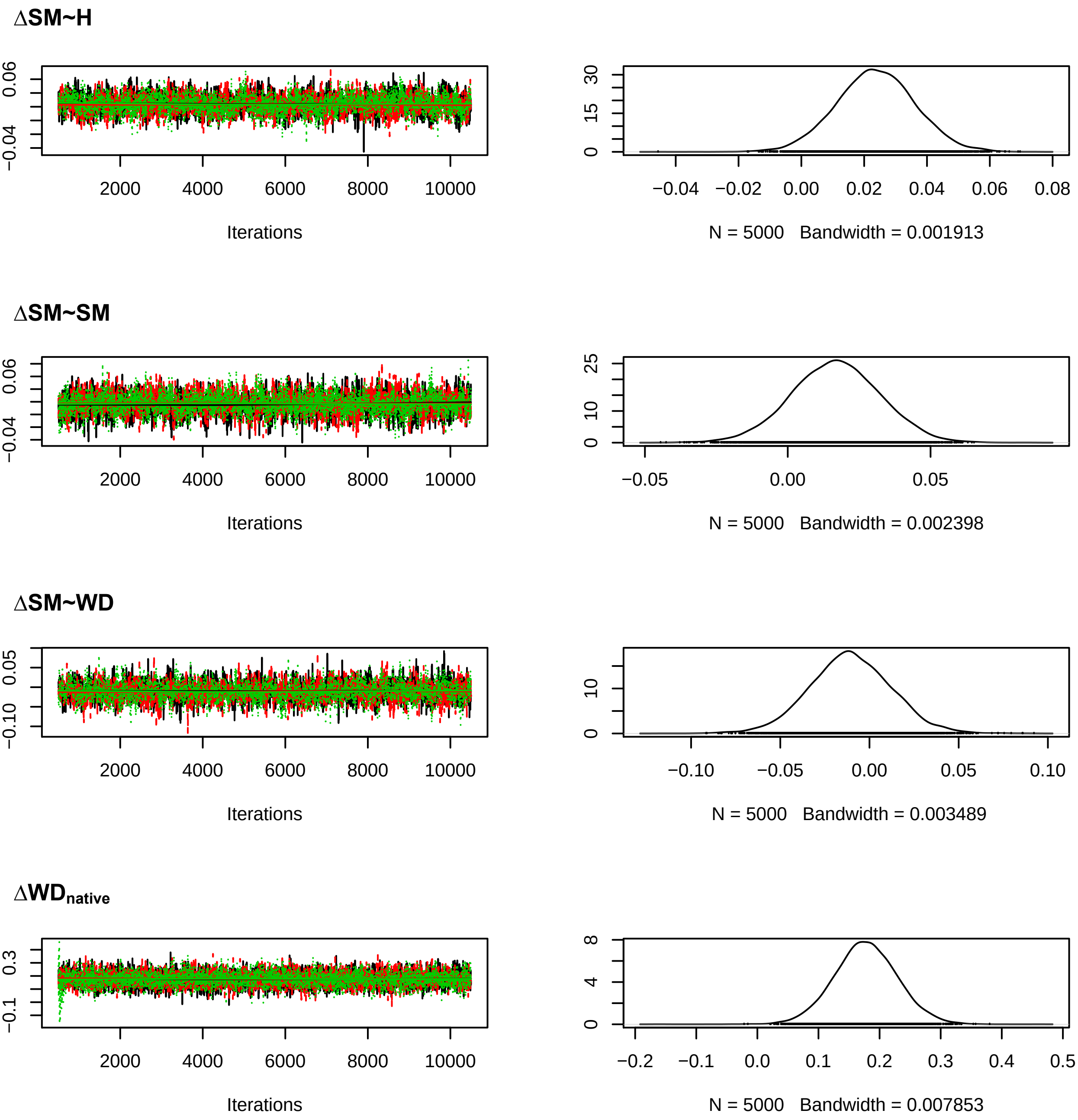
ΔSM~H
Iterations
N = 5000 Bandwidth = 0.001913
ΔSM~SM
Iterations
N = 5000 Bandwidth = 0.002398
ΔSM~WD
Iterations
N = 5000 Bandwidth = 0.003489
ΔWDnative
Iterations
N = 5000 Bandwidth = 0.007853

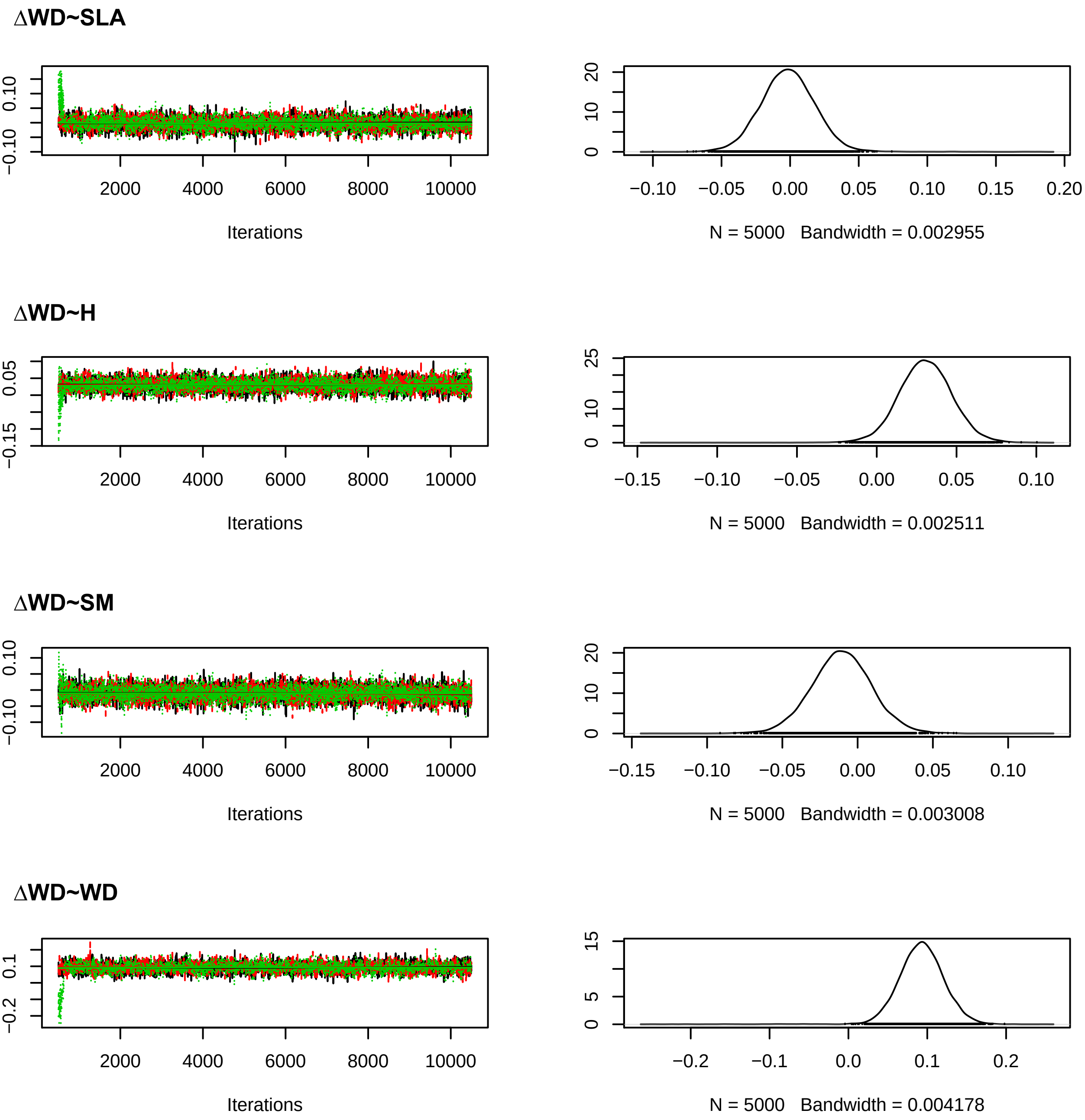
ΔWD~SLA
−0.10
0.10
2000
4000
6000
8000
10000
Iterations
0
10
20
−0.10
−0.05
0.00
0.05
0.10
0.15
0.20
N = 5000 Bandwidth = 0.002955
ΔWD~H
−0.15
0.05
Iterations
0
10
25
−0.15
−0.10
−0.05
0.00
0.05
0.10
N = 5000 Bandwidth = 0.002511
ΔWD~SM
−0.10
0.10
Iterations
0
10
20
N = 5000 Bandwidth = 0.003008
ΔWD~WD
−0.2
0.1
Iterations
0
5
15
−0.2
−0.1
0.0
0.1
0.2
N = 5000 Bandwidth = 0.004178

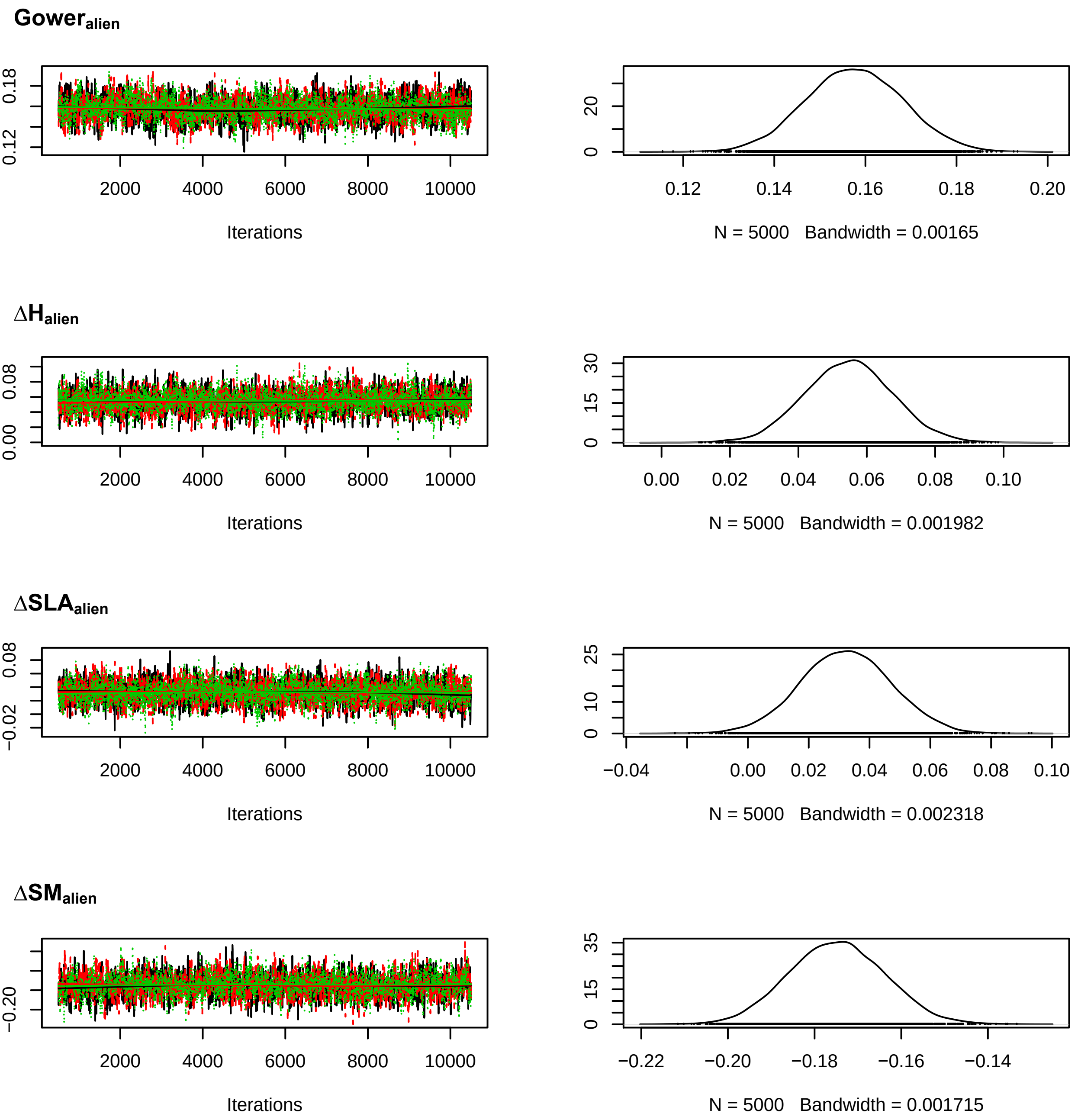
Gower alien
0.12
0.18
2000
4000
6000
8000
10000
Iterations
0
20
0.12
0.14
0.16
0.18
0.20
N = 5000 Bandwidth = 0.00165
ΔH alien
0.00
0.08
Iterations
0
15
30
0.00
0.02
0.04
0.06
0.08
0.10
N = 5000 Bandwidth = 0.001982
ΔSLA alien
−0.02
0.08
Iterations
0
10
25
−0.04
0.00
0.02
0.04
0.06
0.08
0.10
N = 5000 Bandwidth = 0.002318
ΔSM alien
−0.20
Iterations
0
15
35
−0.22
−0.20
−0.18
−0.16
−0.14
N = 5000 Bandwidth = 0.001715

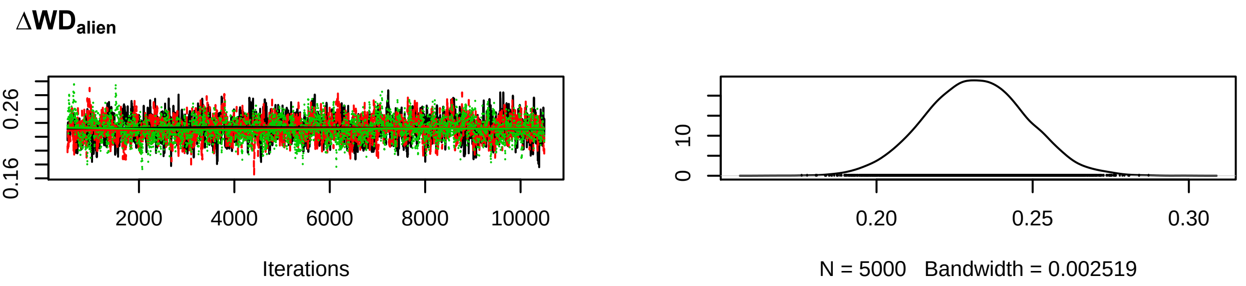
ΔWD alien
0.16
0.26
2000
4000
6000
8000
10000
Iterations
0
10
0.20
0.25
0.30
N = 5000   Bandwidth = 0.002519
