# Supplementary material for: Similar factors underlie tree abundance in forests in native and alien ranges
Source: Glob Ecol Biogeogr. 2019 Dec 1;29(2):281–94. doi: 10.1111/geb.13027 (PMC7006795; doi:10.1111/geb.13027)
Supplement: Supplementary file 3 [file GEB-29-281-s003.docx]

**Appendix S3:** Phylogenetic signal in the random species intercepts, the random species slopes for the effect of the five predictor variables (multivariate dissimilarity and the four trait hierarchy indices) on abundance, and the residuals of the random species intercepts and slopes. The strength of phylogenetic signal was tested using Pagel’s lambda, which varies between 0 (no signal) and 1 (strong signal).

| **Phylogenetic signal tested on:** | **Lambda** | **P-value** |
| --- | --- | --- |
| Random intercept | 0.747 | 0.465 |
| Random slope multivariate dissimilarity | <0.001 | 1.000 |
| Random slope H.hier | <0.001 | 1.000 |
| Random slope SLA.hier | 0.252 | 0.177 |
| Random slope SM.hier | <0.001 | 1.000 |
| Random slope WD.hier | <0.001 | 1.000 |
| Residuals random intercept | 0.861 | 0.160 |
| Residuals random slope multivariate dissimilarity | <0.001 | 1.000 |
| Residuals random slope H.hier | <0.001 | 1.000 |
| Residuals random slope SLA.hier | <0.001 | 1.000 |
| Residuals random slope SM.hier | <0.001 | 1.000 |
| Residuals random slope WD.hier | <0.001 | 1.000 |

**Appendix S4:** Mathematical model description.

To test for effects of dissimilarity (Gower), competitive trait differences (ΔSLA, ΔH, ΔSM, ΔWD), human influence index (HII) and the standardized precipitation and evapotranspiration index (SPEI) on relative dominance, and to test how these effects differ between native and alien ranges as well as between species, we formulated a hierarchical linear regression model. The log-transformed relative dominance (*y_s,p_*) for each occurring combination of focal species *s* and plot *p* was modelled as

*y_s,p_* ~ Normal(*μ_s,p_*, σ^2^)

*μ_s,p_* = ***β****_s_* **∙** ***X****_s,p_* + ***β.inv*** **∙** (***X****_s,p_ ∙ Inv_s,p_*) + *β.spr* ∙ *SPR_p_* + *ε.P_p_*

*ε.P_p_* ~ Normal(0, *σ.P*²)

where ***β****_s_* is a vector of species-specific regression coefficients including the intercept and the effect of each other covariate in the vector ***X****_s,p_* = {1, *Gower_s,p_, ΔSLA_s,p_, ΔH_s,p_, ΔSM_s,p_, ΔWD_s,p_, HII_p_, SPEI_p_*} on relative dominance in the native range. The interaction parameters in ***β.inv*** describe how each of these regression coefficients differs in the invaded range (*Inv_s,p_* = 1 in the invaded range, zero native range). Additionally, we included plot-level random effects (*ε.P*) and an effect of the species richness (SPR) on each plot because high richness (which is correlated with large plot size) decreases the average relative dominance of species and can influence dissimilarity indices. In order to describe trait-dependent interspecific variation, we then formulated separate regression models for each type of intercept and slope parameter in ***β***. The parameter *β_s,k_* , i.e. the response of species *s* to the *k^th^* covariate in ***X****_s,p_*, is thus modelled as

*β_s,k_* ~ Normal(*μ.β_s,k_*, *σ.S_k_*²)

*μ.β_s,k_* = *α_k_* + *γ.sla_k_* ∙ *SLA_s_* + *γ.h_k_* ∙ *H_s_* + *γ.sm_k_* ∙ *SM_s_* + *γ.wd_k_* ∙ *WD_s_*

Hence, for example *α_1_* is the overall model intercept, *α_2_* is the average effect of *Gower* on relative dominance in the native range (“Gower_native_” in Fig. 2), *α_2_* + *β.inv_2_* is the average effect of *Gower* on relative dominance in the invaded range (“Gower_alien_” in Fig. 2) and *γ.sla_2_* describes how species SLA affects this response to *Gower* (“*Gower* ~ *SLA*” in Fig. 3). We did not include an interaction between these species-specific slope differences and range status (*Inv*), because relationships of fixed effects on dominance showed generally little differences between native and alien range (Fig. 2). All covariates were scaled prior to analysis by subtracting the mean and dividing by the standard deviation.
