# Supplementary material for: Similar factors underlie tree abundance in forests in native and alien ranges
Source: Glob Ecol Biogeogr. 2019 Dec 1;29(2):281–94. doi: 10.1111/geb.13027 (PMC7006795; doi:10.1111/geb.13027)
Supplement: Supplementary file 4 [file GEB-29-281-s004.pdf]

Appendix S5 - R script

```
### 1) Build model
sink('Model.txt')
cat('
    model {
    ### likelihood
    for (i in 1:i.max) {
    Ln.Relative.woody.cover[i] ~ dnorm(mu[i],tau)
    mu[i] <-  alpha[i] + gower[i]*gower.sc[i] + sla.hier[i]*sla.hier.sc[i] + 
h.hier[i]*h.hier.sc[i] +   sm.hier[i]*sm.hier.sc[i] + wd.hier[i]*wd.hier.sc[i] 
+ HII[i]*HII_prc.sc[i] + SPEI[i]*SPEI12.sc[i] + SPR*sp.richness.sc[i]
    # alpha[i] <- alpha.mu + Sp.rand.int[SP[i]] + PLOT.rand.int[PLOT[i]]
    alpha[i] <- alpha.Sp[SP[i]] + alpha.alien * is.alien[i] + 
PLOT.rand.int[PLOT[i]]
    gower[i] <- gower.Sp[SP[i]] + gower.alien * is.alien[i]
    sla.hier[i] <- sla.hier.Sp[SP[i]] + sla.hier.alien * is.alien[i]
    h.hier[i] <- h.hier.Sp[SP[i]] + h.hier.alien * is.alien[i]
    sm.hier[i] <- sm.hier.Sp[SP[i]] + sm.hier.alien * is.alien[i]
    wd.hier[i] <- wd.hier.Sp[SP[i]] + wd.hier.alien * is.alien[i]
    HII[i] <- HII.Sp[SP[i]] + HII.alien * is.alien[i]
    SPEI[i] <- SPEI.Sp[SP[i]] + SPEI.alien * is.alien[i]
    }

    for(k in 1: n.PLOT){
    ### random intercepts for plots
        PLOT.rand.int[k] ~ dnorm(0, tau.PLOT)
    }

    for(l in 1: n.SP){
    ### random intercepts for species
    alpha.Sp[l] ~ dnorm(mu.alpha[l], tau.SP)

    ### random slopes for species
    gower.Sp[l] ~ dnorm(mu.gower[l], tau.gower)
    sla.hier.Sp[l] ~ dnorm(mu.sla.hier[l], tau.sla.hier)
    h.hier.Sp[l] ~ dnorm(mu.h.hier[l], tau.h.hier)
    sm.hier.Sp[l] ~ dnorm(mu.sm.hier[l], tau.sm.hier)
    wd.hier.Sp[l] ~ dnorm(mu.wd.hier[l], tau.wd.hier)
    HII.Sp[l] ~ dnorm(mu.HII[l], tau.HII)
    SPEI.Sp[l] ~ dnorm(mu.SPEI[l], tau.SPEI)
    }

    mu.alpha <- X.Sp %*% beta.alpha
    mu.gower <- X.Sp %*% beta.gower
    mu.sla.hier <- X.Sp %*% beta.sla.hier
    mu.h.hier <- X.Sp %*% beta.h.hier
    mu.sm.hier <- X.Sp %*% beta.sm.hier
    mu.wd.hier <- X.Sp %*% beta.wd.hier
    mu.HII <- X.Sp %*% beta.HII
    mu.SPEI <- X.Sp %*% beta.SPEI
```

```
    ### priors
    alpha.mu ~ dnorm(0,0.001)

    # priors for categorical fixed effects for both levels
    alpha.alien ~ dnorm(0, 0.001)
    gower.alien ~ dnorm(0, 0.001)
    sla.hier.alien  ~ dnorm(0, 0.001)
    h.hier.alien ~ dnorm(0, 0.001)
    sm.hier.alien ~ dnorm(0, 0.001)
    wd.hier.alien ~ dnorm(0, 0.001)
    HII.alien ~ dnorm(0, 0.001)
    SPEI.alien ~ dnorm(0, 0.001)

    for(t in 1:n.trait){
    beta.alpha[t] ~ dnorm(0,0.001)
    beta.gower[t] ~ dnorm(0,0.001)
    beta.sla.hier[t] ~ dnorm(0,0.001)
    beta.h.hier[t] ~ dnorm(0,0.001)
    beta.sm.hier[t]~ dnorm(0,0.001)
    beta.wd.hier[t] ~ dnorm(0,0.001)
    beta.HII[t] ~ dnorm(0,0.001)
    beta.SPEI[t] ~ dnorm(0,0.001)
    }
    SPR ~ dnorm(0,0.001)
    tau <- 1/(sigma*sigma)
    sigma ~ dunif(0,100)
    tau.PLOT <- 1/(sigma.PLOT*sigma.PLOT)
    sigma.PLOT ~ dunif(0,100)
    tau.SP <- 1/(sigma.SP*sigma.SP)
    sigma.SP ~ dunif(0,100)
    tau.gower <- 1/(sigma.gower*sigma.gower)
    sigma.gower ~ dunif(0,100)
    tau.sla.hier <- 1/(sigma.sla.hier*sigma.sla.hier)
    sigma.sla.hier ~ dunif(0,100)
    tau.h.hier <- 1/(sigma.h.hier*sigma.h.hier)
    sigma.h.hier ~ dunif(0,100)
    tau.sm.hier <- 1/(sigma.sm.hier*sigma.sm.hier)
    sigma.sm.hier ~ dunif(0,100)
    tau.wd.hier <- 1/(sigma.wd.hier*sigma.wd.hier)
    sigma.wd.hier ~ dunif(0,100)
    tau.HII <- 1/(sigma.HII*sigma.HII)
    sigma.HII ~ dunif(0,100)
    tau.SPEI <- 1/(sigma.SPEI*sigma.SPEI)
    sigma.SPEI ~ dunif(0,100)
    }
    ',fill=TRUE)
sink()

# Create a model design matrix for the desired trait effects
X.Sp <- model.matrix(~ SLA.mean.sc + PlantHeight.mean.sc + SeedMass.mean.sc +
StemDens.mean.sc, data = Sp.data)
```

```
### 2) Set up a list that contains all the necessary data
Data.subs = list(Ln.Relative.woody.cover = Data$Ln.Relative.woody.cover,
gower.sc = Data$gower.sc,  sla.hier.sc = Data$sla.hier.sc, h.hier.sc =
Data$h.hier.sc, sm.hier.sc = Data$sm.hier.sc, wd.hier.sc = Data$wd.hier.sc,
is.alien = as.numeric(Data$range == 'alien'),
                 HII_prc.sc = Data$HII_prc.sc, SPEI12.sc = Data$SPEI12.sc,
sp.richness.sc = Data$sp.richness.sc, X.Sp = X.Sp, n.trait = ncol(X.Sp),  i.max
= nrow(Data), n.PLOT = max(Data$plot.nr), n.SP = max(Data$sp.nr),
SP=Data$sp.nr, PLOT=Data$plot.nr)

### 3) Specify a function to generate initial values for the parameters
inits.fn.subs <- function() list(beta.alpha = rnorm(ncol(X.Sp)),
                                 beta.gower = rnorm(ncol(X.Sp)), beta.sla.hier
= rnorm(ncol(X.Sp)), beta.h.hier = rnorm(ncol(X.Sp)), beta.sm.hier =
rnorm(ncol(X.Sp)),
                                 beta.HII = rnorm(ncol(X.Sp)), beta.SPEI =
rnorm(ncol(X.Sp)),
                                 alpha.alien = rnorm(1),
                                 gower.alien = rnorm(1), sla.hier.alien =
rnorm(1), h.hier.alien = rnorm(1), sm.hier.alien = rnorm(1),
                                 HII.alien = rnorm(1), SPEI.alien = rnorm(1),
                                 SPR = rnorm(1),
                                 sigma = runif(1,1,100), sigma.PLOT =
runif(1,1,100), sigma.SP = runif(1,1,100), sigma.gower = runif(1,1,100),
                                 sigma.sla.hier = runif(1,1,100), sigma.h.hier
= runif(1,1,100), sigma.sm.hier = runif(1,1,100),
                                 sigma.wd.hier = runif(1,1,100), sigma.HII =
runif(1,1,100), sigma.SPEI = runif(1,1,100))


library(snowfall)

n.chains = 3
n.iter = 10000
n.thin = 2

coda.samples.wrapper <- function(j)
{
  temp.model = jags.model("Model.txt",
                          inits=list(.RNG.name="base::Wichmann-Hill",
.RNG.seed=j),
                          data=Data.subs, n.chains=1, n.adapt=500)
  coda.samples(temp.model, c("beta.gower", "beta.sla.hier",
"beta.h.hier","beta.sm.hier" , "beta.wd.hier", "beta.HII", "beta.SPEI",
                             "gower.alien", "sla.hier.alien",
"h.hier.alien","sm.hier.alien" , "wd.hier.alien", "HII.alien", "SPEI.alien",
                             "SPR", "alpha.alien", "beta.alpha"),
n.iter=n.iter, thin=n.thin)
}

# open a cluster
cl <- makeCluster(n.chains, "SOCK")
```

```
# Make sure the rjags library is loaded in each worker
clusterEvalQ(cl, library(rjags))
# Send data to workers, then fit models.
clusterExport(cl, list("Data.subs","n.iter","n.thin")) # variables that will be
sent to the cluster
par.samples = clusterApply(cl, 1:n.chains, coda.samples.wrapper) # applies the
coda sampling and provides list of 3 chains as output
# Reorganize 'par.samples' so that it is recognizeable as an 'mcmc.list' object
for(i in 1:length(par.samples)) { par.samples12[[i]] <- par.samples12[[i]][[1]]
}
class(par. samples) <- "mcmc.list"
stopCluster(cl)

# convergence check
gelman.stat.subs <- gelman.diag(par. samples)
```
