# Supplementary material for: Similar factors underlie tree abundance in forests in native and alien ranges
Source: Glob Ecol Biogeogr. 2019 Dec 1;29(2):281–94. doi: 10.1111/geb.13027 (PMC7006795; doi:10.1111/geb.13027)
Supplement: Supplementary file 5 [file GEB-29-281-s005.docx]

**Appendix S6:** Relative abundance distribution in the native (black) and alien (red) range across all species. The relative abundance was divided into 20 bins (a width of 0.05 relative abundance each), and per bin the proportion of the total frequency was calculated by dividing the frequency by the total number of occurrences in the corresponding range. We use the proportional instead of absolute frequency to show the curves of the native and alien range along the same axis.


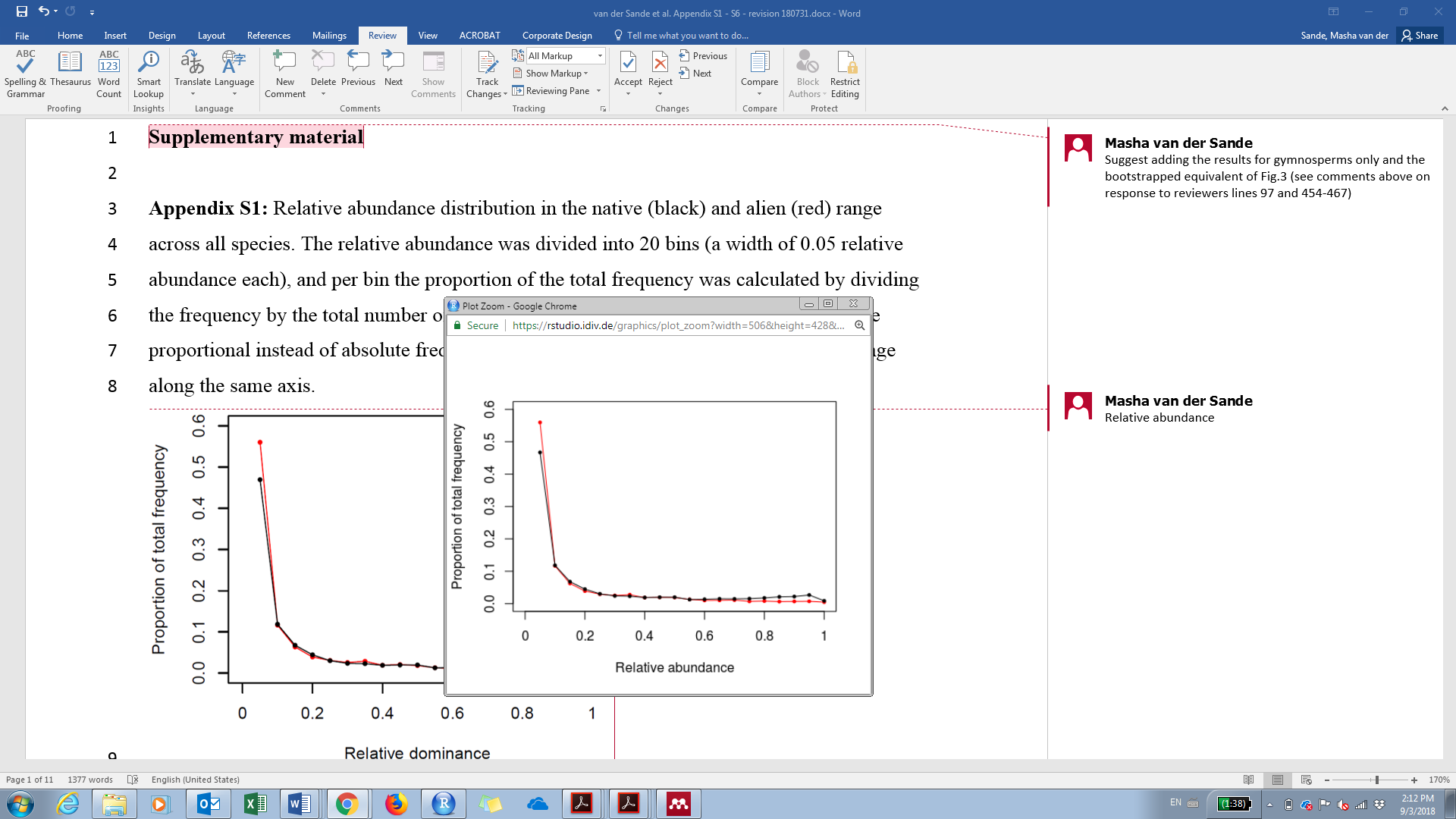


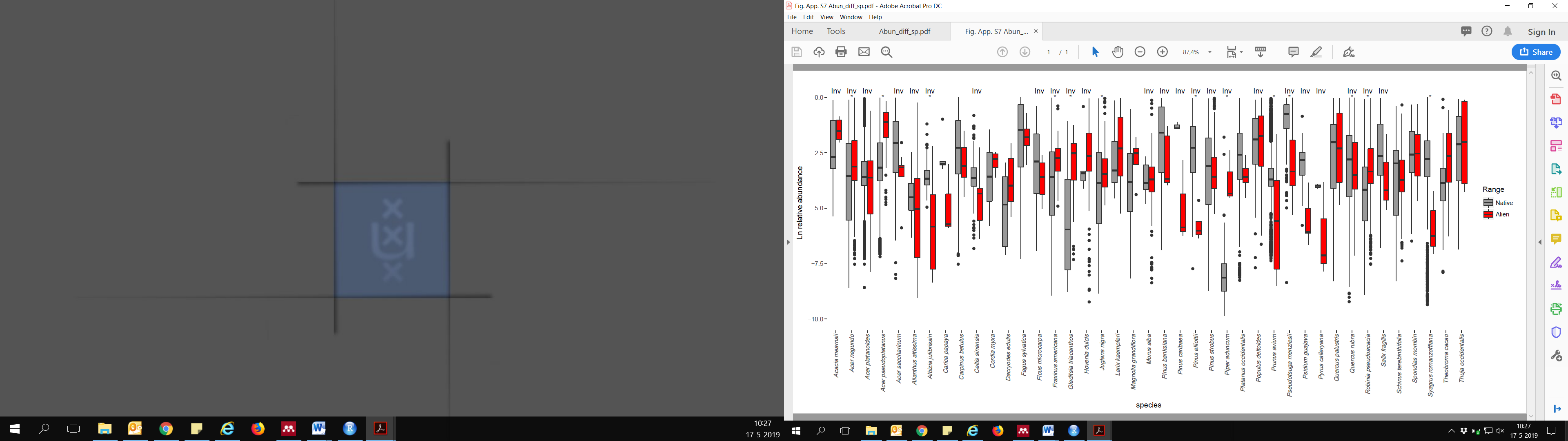


**Appendix S7:** Differences in relative abundance between the native (grey) and alien (red) range of each species. Species that have been classified as invasive are indicated by “Inv”. Significant differences between native and alien range are indicated by “*”.

| a) | b) |
| --- | --- |
| 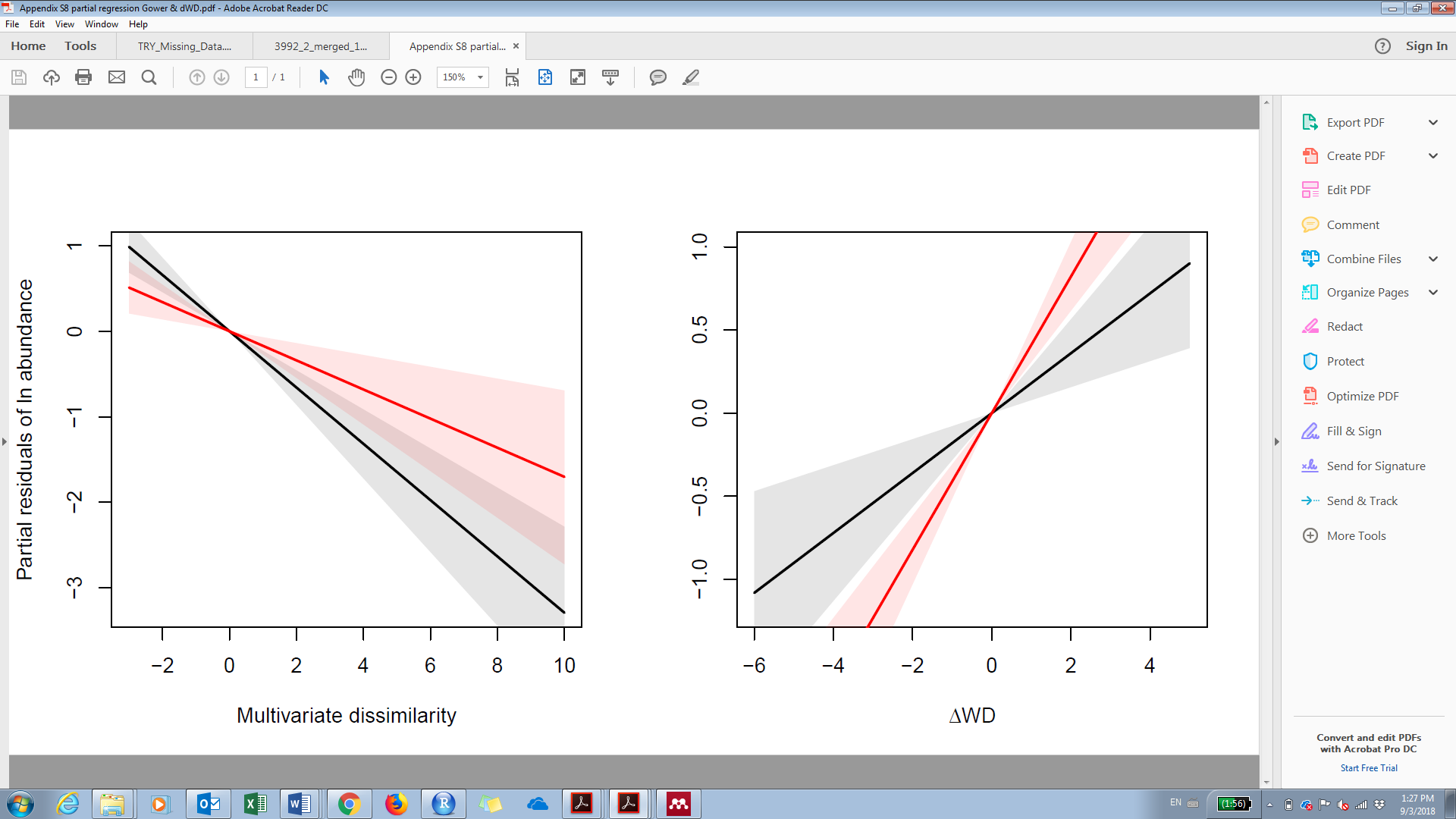 | |

**Appendix S8:** Partial regression results of multivariate dissimilarity (a) and competitive differences in wood density (b; ∆WD) on ln-transformed relative abundance. Multivariate dissimilarity and ∆WD are scaled by subtracting the mean and dividing by the standard deviation. The regression coefficients correspond with results in Fig 3. Black lines refer to the native range and red lines to the alien range.

| a) Invasive species | b) Non-invasive species |
| --- | --- |
| 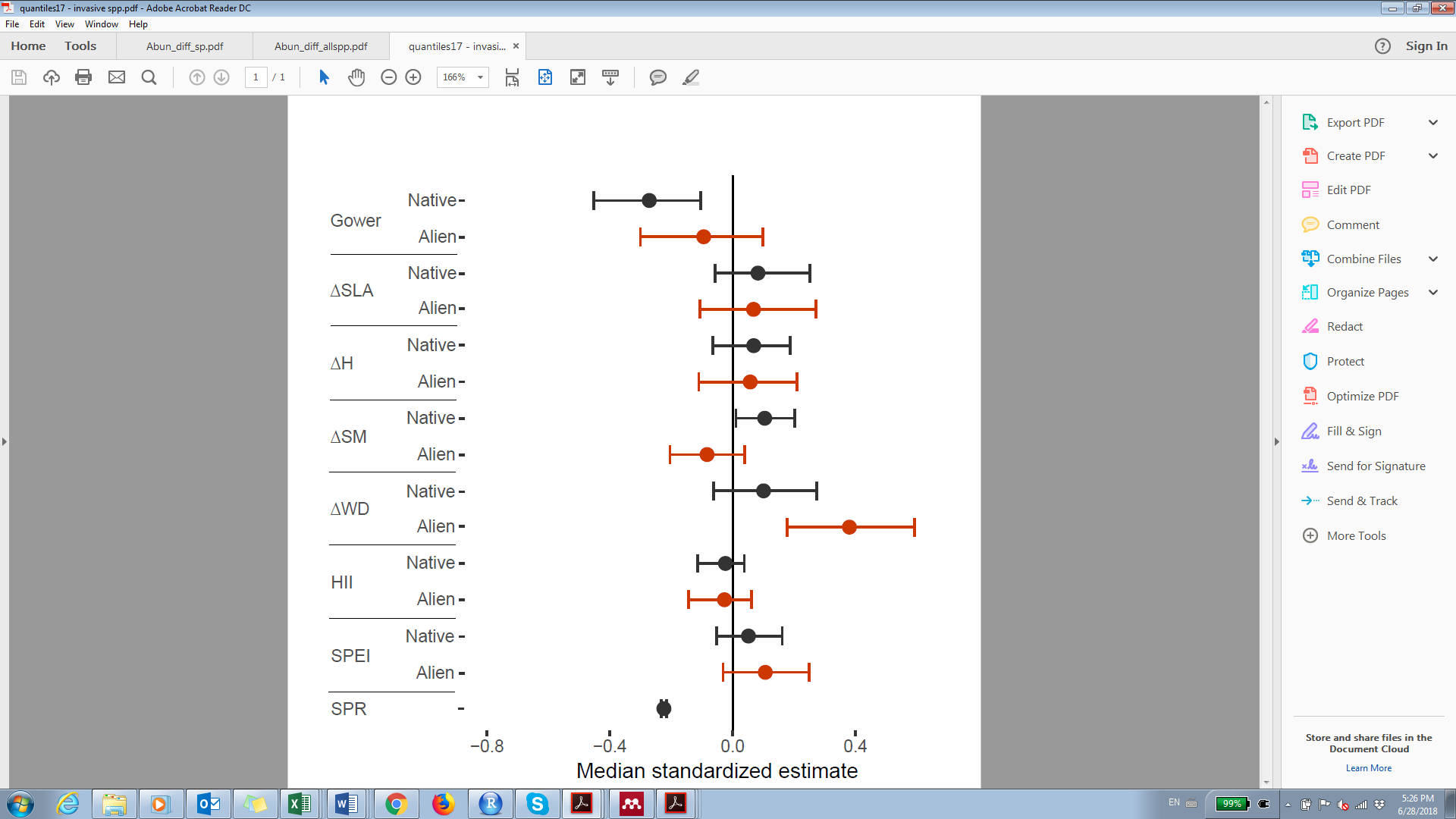 | 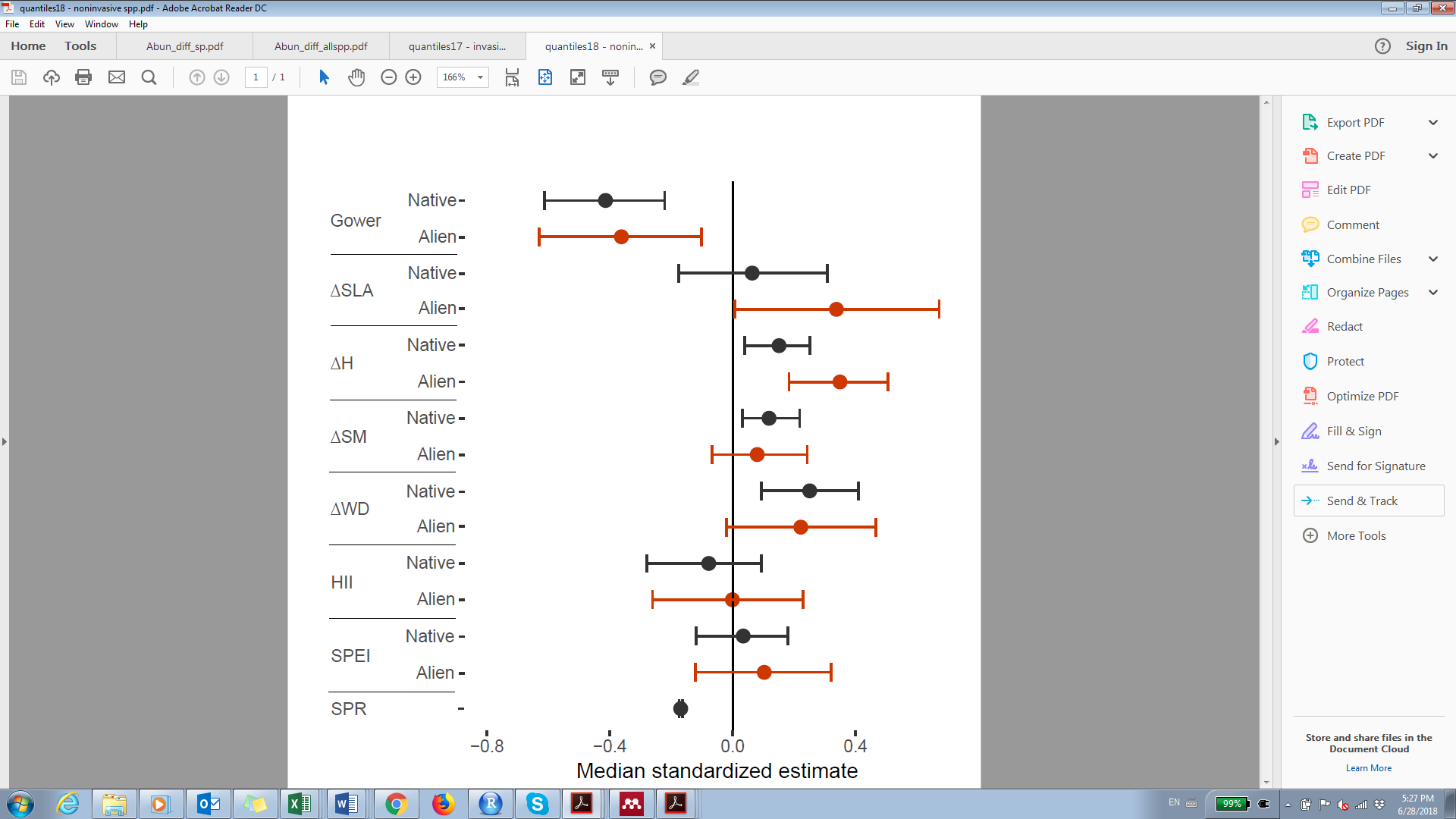 |

**Appendix S9**: Results for a) 24 invasive species (species indicated by “Inv” in Appendix S2) and b) 17 non-invasive species separately, showing the effects of Gower trait dissimilarity, competitive trait differences in specific leaf area (∆SLA), adult height (∆H), seed mass (∆SM) and wood density (∆WD), human influence index (HII), standardized precipitation and evapotranspiration index (SPEI) and species richness (SPR) on relative abundance in the native (black) and alien (red) range. Analyes are similar to analyses in the main text (Fig. 4).

| a) | b) | c) |
| --- | --- | --- |
| 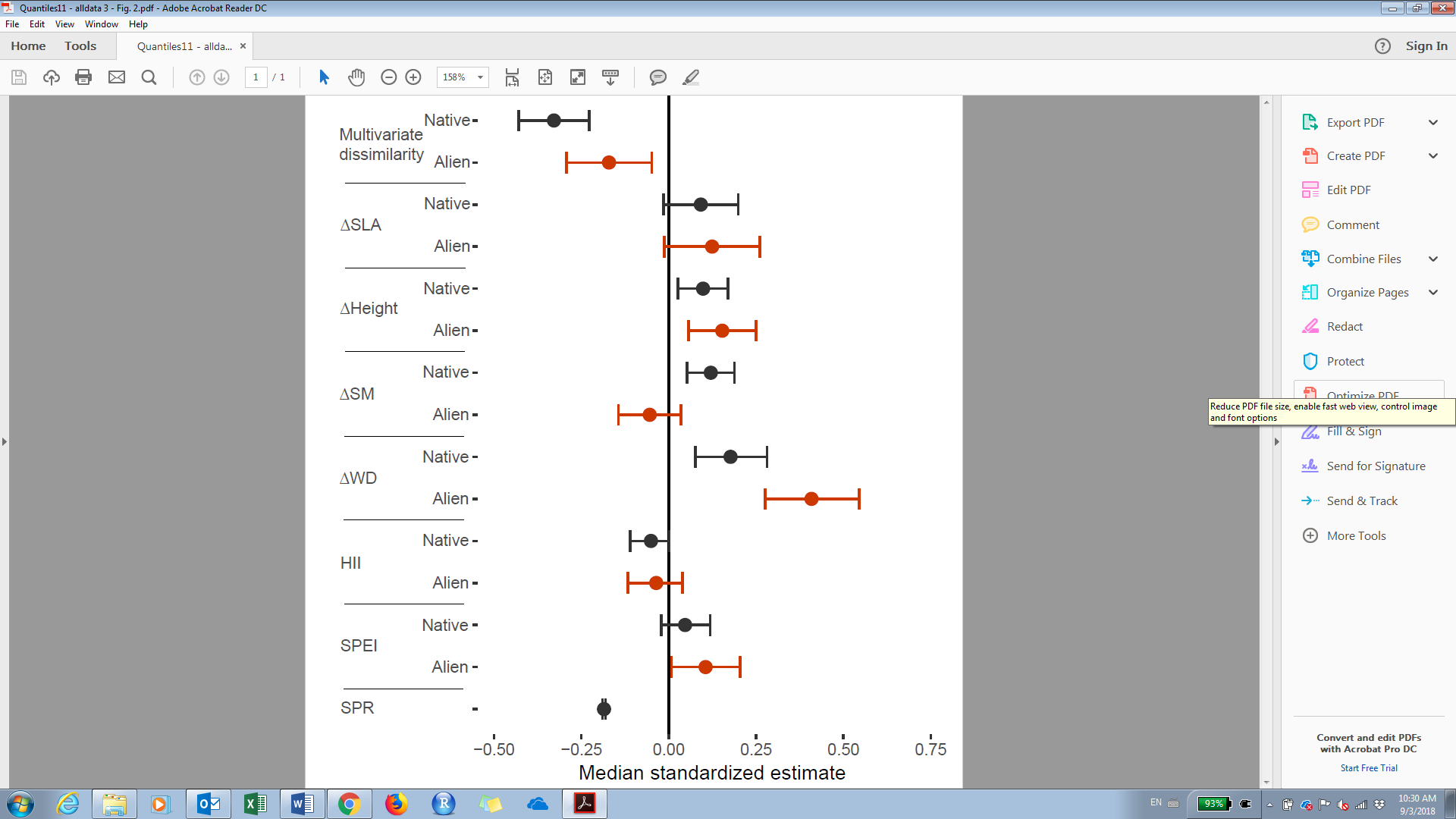 | 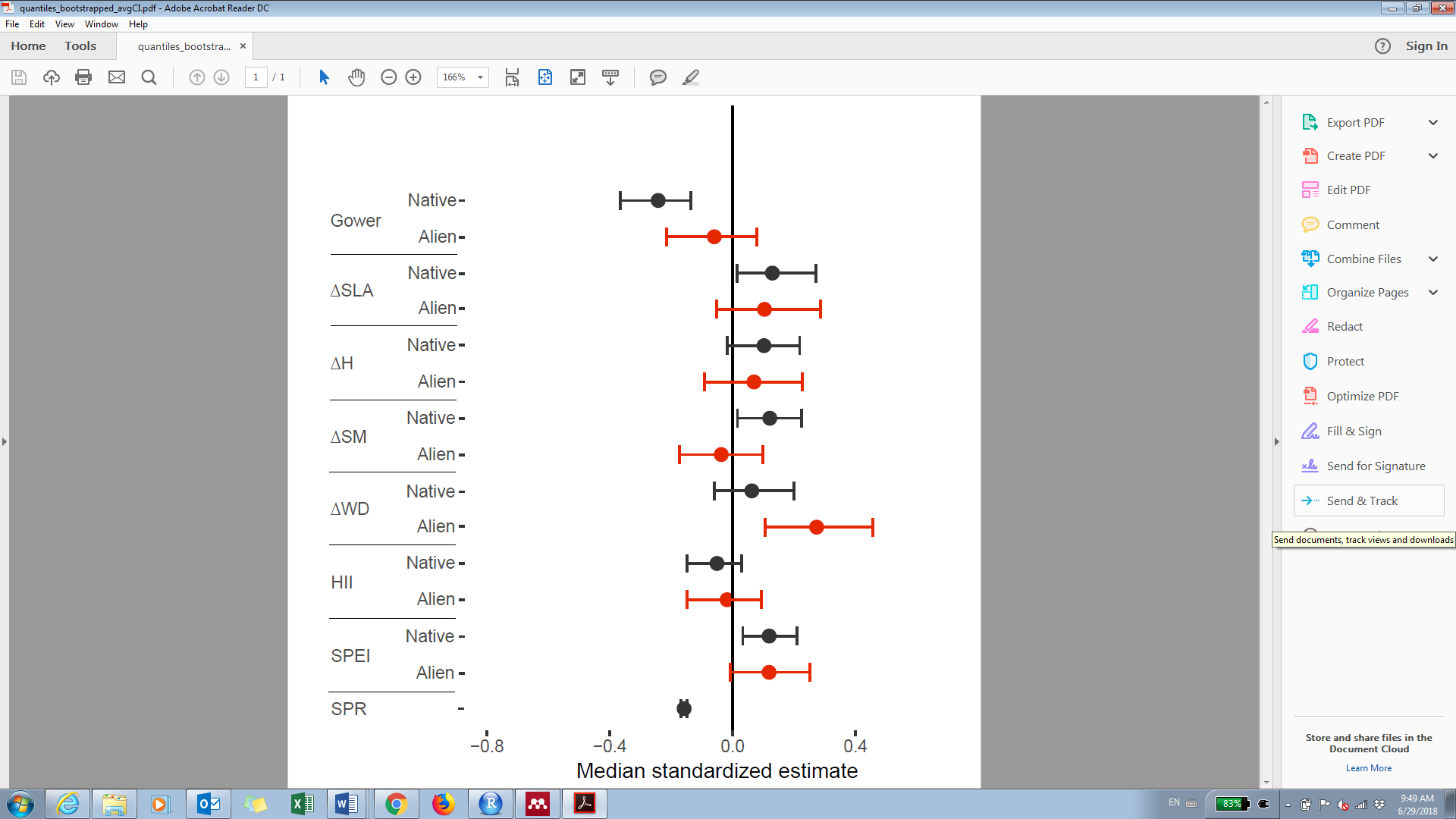 | 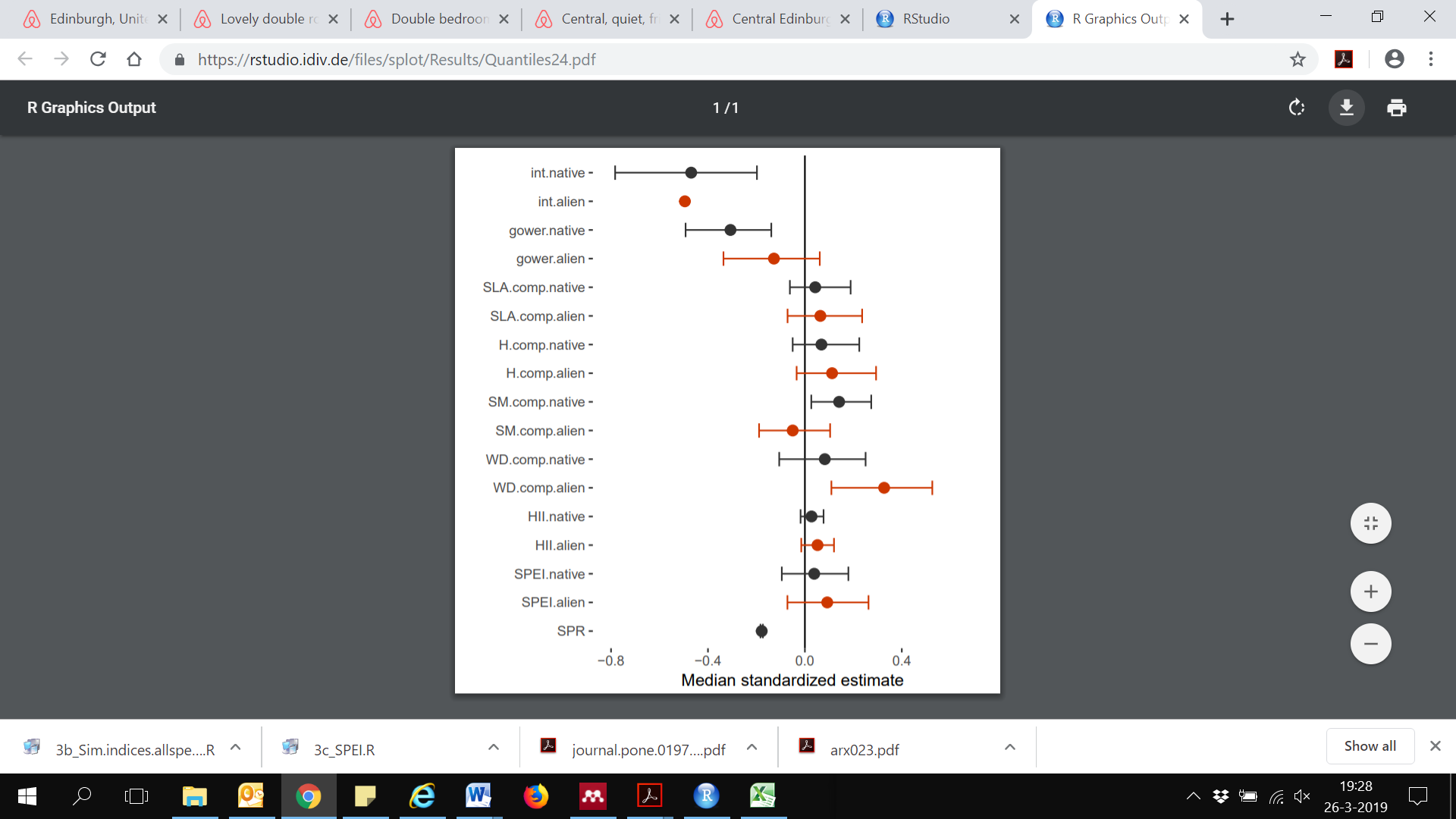 |

**Appendix S10:** Effects of trait dissimilarity, competitive trait differences and environmental conditions on local species abundance in their native (black) and alien (red) range. Figure a) shows the original results (same as Fig. 3), and figures b) and c) show results based on a more balanced dataset. Figure b) was based on 100 bootstraps selecting an equal number of plots in the native and alien range per species, and figure c) was based on 15 species that had at least 50 plots in each range.


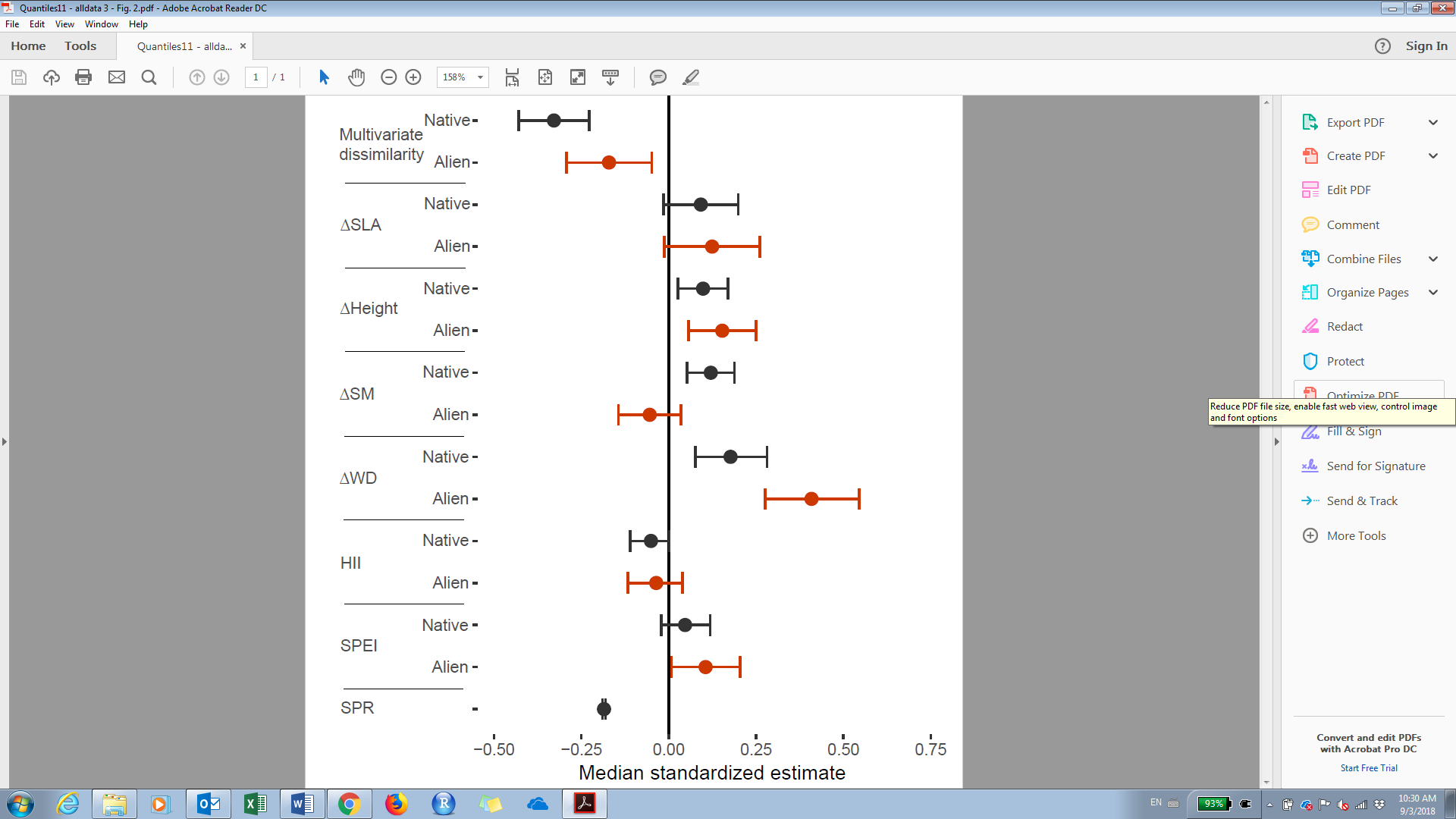

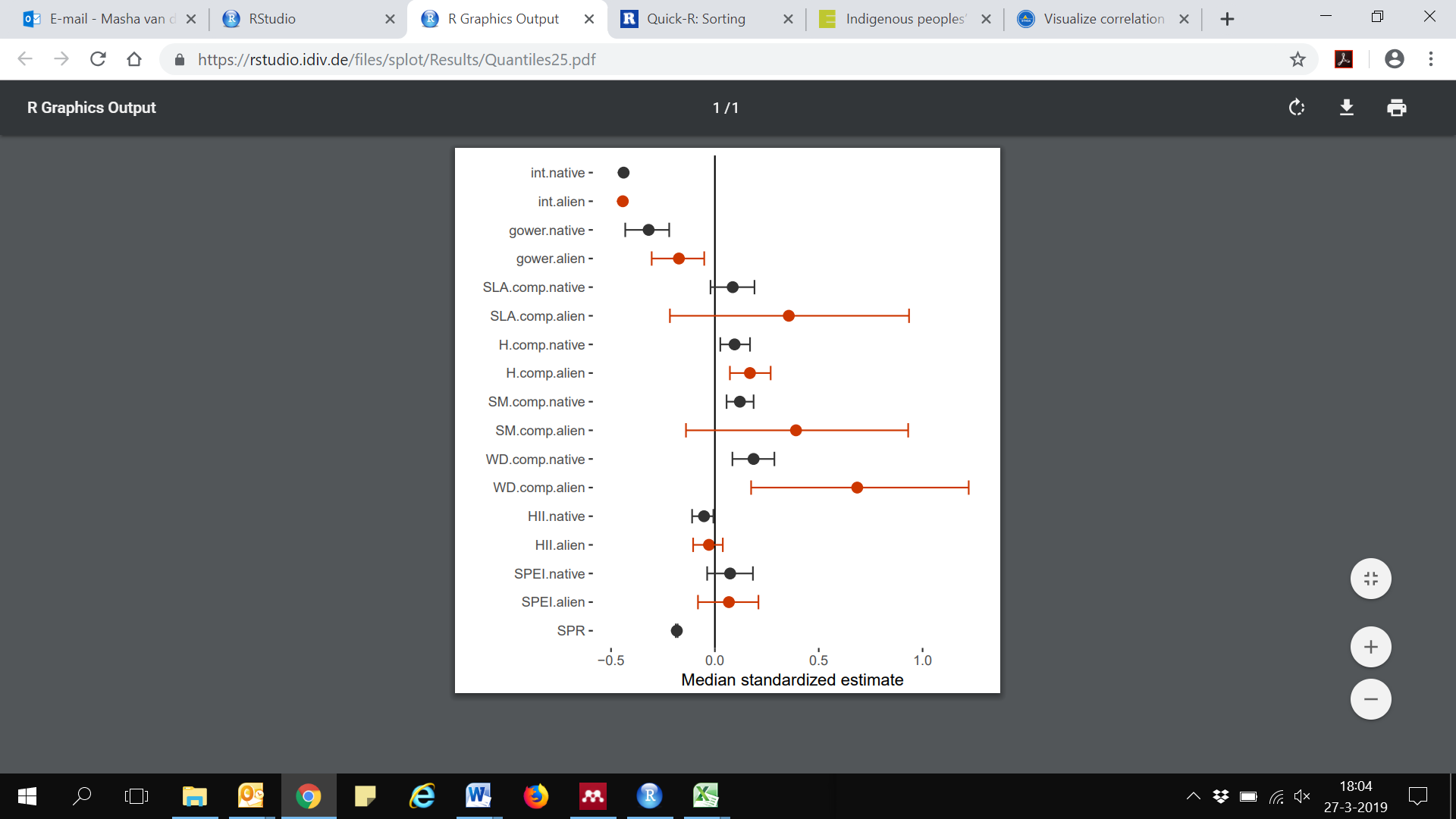


**Appendix S11:** Effects of trait dissimilarity, competitive trait differences and environmental conditions on local species abundance in their native (black) and alien (red) range, with original SPEI (standardized precipitation and evapotranspiration index, Fig. 3) changed for dissimilarity between local SPEI and species optimal SPEI. Species optimal SPEI was calculated as the average SPEI at which the species occurred in its native range. SPEI dissimilarity values were all changed to absolute values, so that high values indicate high dissimilarity to optimal SPEI value, and values close to zero indicate high similarity to optimal SPEI value.

| a) | b) |
| --- | --- |
| 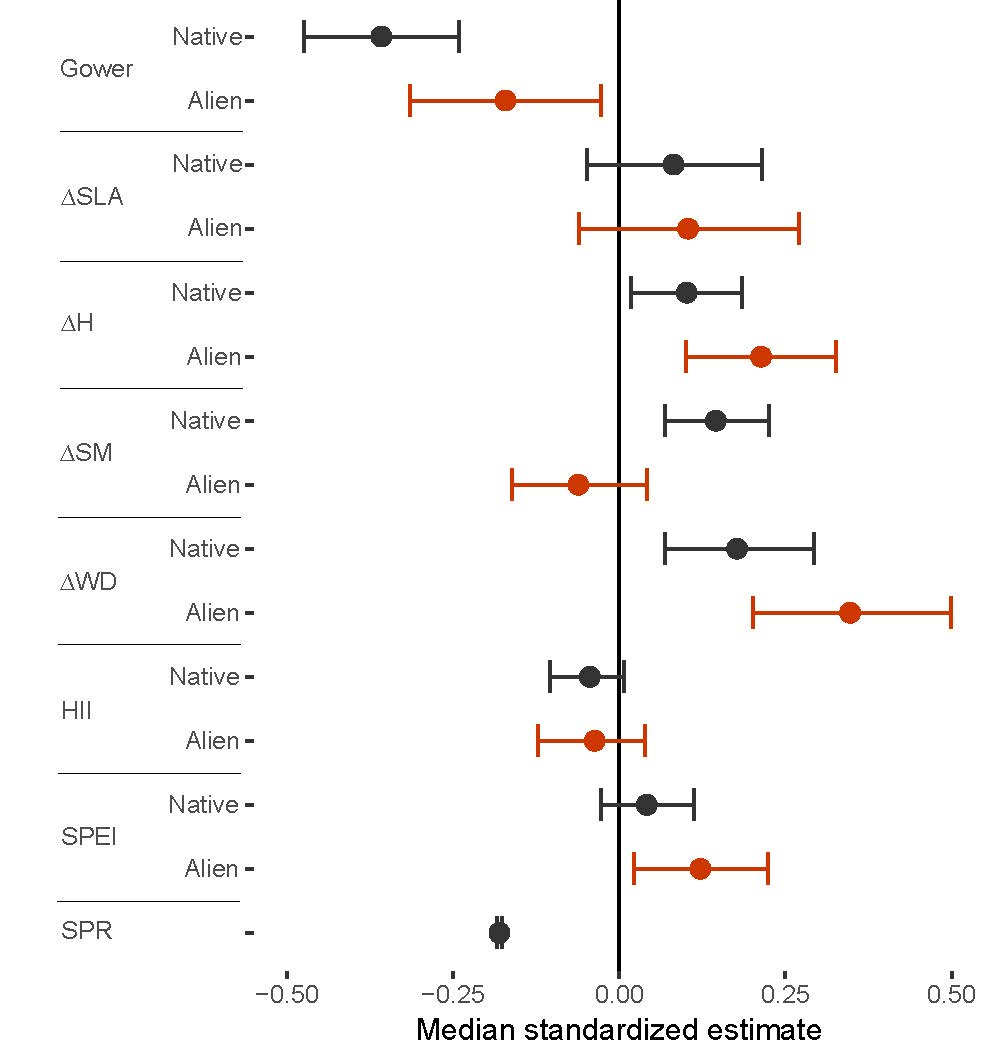 | 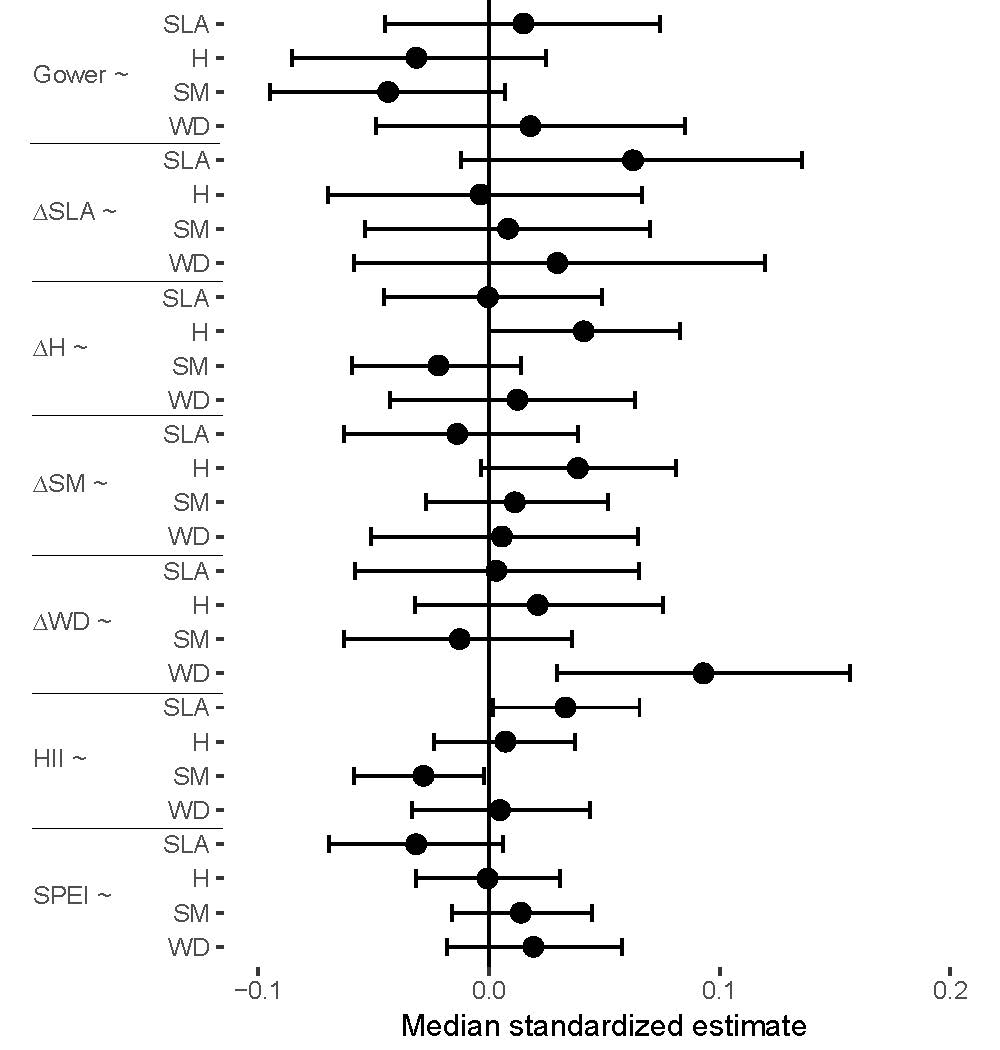 |

**Appendix S12**: Results after excluding gymnosperms and non-tree species (*Carica papaya* and *Syagrus romanzoffiana*). Figure a) shows the effects of Gower trait dissimilarity, competitive trait differences in specific leaf area (∆SLA), adult height (∆H), seed mass (∆SM) and wood density (∆WD), human influence index (HII), standardized precipitation and evapotranspiration index (SPEI) and species richness (SPR) on relative abundance in the native (black) and alien (red) range. Figure b) shows how traits (SLA, H, SM and WD) influence species-specific differences in the slopes of the relationships of predictors (Gower, ∆SLA, ∆H, ∆SM, ∆WD, HII and SPEI) with relative abundance. Analyes are similar to analyses in the main text (Fig. 3), and based on 33 species and 222,000 plots (208,691 in the native and 13,309 in the alien range).


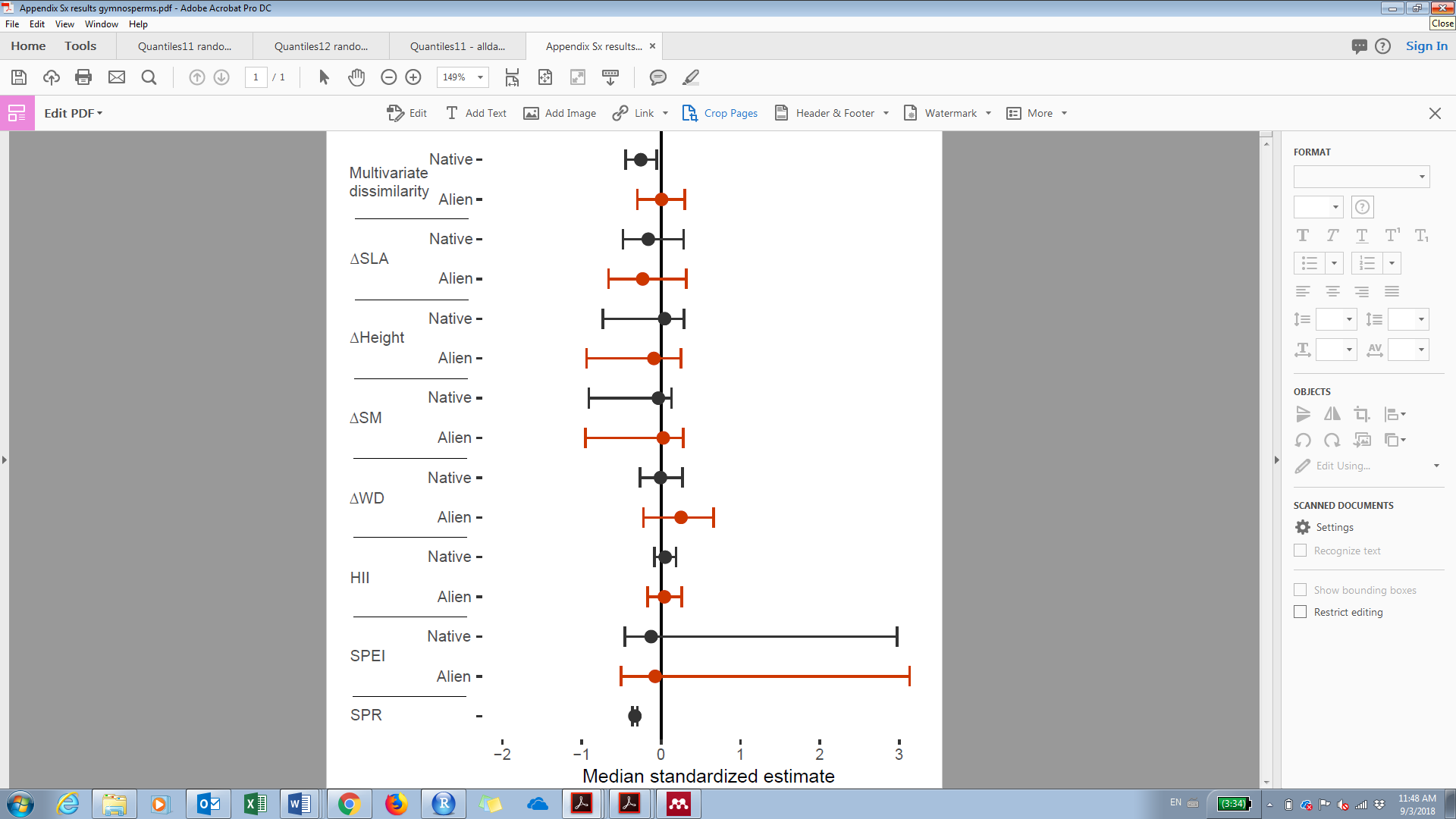


**Appendix S13:** Results for gymnosperms only. The analyses are similar to those presented in the main manuscript, but effects of traits on random slopes of species were excluded because of a low number (7) of species included.

**Appendix S14:** Quantile estimates for the effects of multivariate dissimilarity (Mult.diss.), competitive trait differences of specific leaf area (∆SLA), adult height (∆H), seed mass (∆SM) and wood density (∆WD), human influence index (HII), standardized precipitation and evapotranspiration index (SPEI), and species richness (SPR) in native and alien range on relative species’ abundance. The 50% quantile (i.e. the median standardized effect size) and the 2.5% and 97.5% quantiles (i.e. the 95% credible intervals) in a) correspond with Fig. 3 in the main text, and the quantiles in b) correspond with Fig. 4 in the main text. Note that the intercept effects are not shown in Fig. 3 and 4.

|  | **Predictor variable** | **2.5%** | **25%** | **50%** | **75%** | **97.5%** |
| --- | --- | --- | --- | --- | --- | --- |
| a) | Intercept_native_ | -0.697 | -0.486 | -0.383 | -0.280 | -0.074 |
|  | Intercept_alien_ | -0.060 | -0.038 | -0.027 | -0.016 | 0.006 |
|  | Mult.diss._native_ | -0.430 | -0.362 | -0.328 | -0.294 | -0.227 |
|  | Mult.diss._alien_ | 0.137 | 0.150 | 0.158 | 0.165 | 0.179 |
|  | ∆SLA_native_ | -0.015 | 0.056 | 0.092 | 0.128 | 0.199 |
|  | ∆SLA_alien_ | 0.003 | 0.022 | 0.032 | 0.042 | 0.062 |
|  | ∆H_native_ | 0.026 | 0.075 | 0.098 | 0.122 | 0.169 |
|  | ∆H_alien_ | 0.030 | 0.046 | 0.055 | 0.063 | 0.081 |
|  | ∆SM_native_ | 0.052 | 0.098 | 0.120 | 0.143 | 0.188 |
|  | ∆SM_alien_ | -0.196 | -0.182 | -0.175 | -0.167 | -0.153 |
|  | ∆WD_native_ | 0.076 | 0.144 | 0.177 | 0.212 | 0.281 |
|  | ∆WD_alien_ | 0.200 | 0.221 | 0.232 | 0.243 | 0.264 |
|  | HII_native_ | -0.110 | -0.070 | -0.051 | -0.033 | 0.001 |
|  | HII_alien_ | -0.008 | 0.007 | 0.015 | 0.023 | 0.038 |
|  | SPEI_native_ | -0.021 | 0.023 | 0.047 | 0.071 | 0.119 |
|  | SPEI_alien_ | 0.030 | 0.049 | 0.059 | 0.068 | 0.086 |
|  | SPR | -0.190 | -0.187 | -0.185 | -0.184 | -0.181 |
| b) | Intercept~SLA | -0.191 | -0.109 | -0.068 | -0.028 | 0.051 |
|  | Intercept~H | -0.059 | 0.009 | 0.043 | 0.078 | 0.149 |
|  | Intercept~SM | -0.101 | -0.020 | 0.021 | 0.062 | 0.147 |
|  | Intercept~WD | -0.235 | -0.123 | -0.065 | -0.006 | 0.116 |
|  | Mult.diss.~SLA | -0.036 | -0.012 | 0.001 | 0.012 | 0.037 |
|  | Mult.diss.~H | -0.048 | -0.027 | -0.017 | -0.007 | 0.014 |
|  | Mult.diss.~SM | -0.080 | -0.053 | -0.039 | -0.026 | 0.000 |
|  | Mult.diss.~WD | -0.054 | -0.019 | -0.001 | 0.016 | 0.051 |
|  | ∆SLA~SLA | 0.016 | 0.045 | 0.059 | 0.073 | 0.102 |
|  | ∆SLA~H | -0.012 | 0.011 | 0.023 | 0.035 | 0.058 |
|  | ∆SLA~SM | -0.052 | -0.022 | -0.006 | 0.009 | 0.040 |
|  | ∆SLA~WD | -0.037 | 0.007 | 0.029 | 0.050 | 0.093 |
|  | ∆H~SLA | -0.026 | -0.008 | 0.000 | 0.010 | 0.029 |
|  | ∆H~H | -0.008 | 0.007 | 0.014 | 0.022 | 0.037 |
|  | ∆H~SM | -0.039 | -0.020 | -0.011 | -0.002 | 0.017 |
|  | ∆H~WD | -0.022 | 0.006 | 0.020 | 0.033 | 0.059 |
|  | ∆SM~SLA | -0.017 | 0.002 | 0.013 | 0.023 | 0.044 |
|  | ∆SM~H | -0.001 | 0.015 | 0.024 | 0.032 | 0.048 |
|  | ∆SM~SM | -0.014 | 0.006 | 0.017 | 0.027 | 0.047 |
|  | ∆SM~WD | -0.056 | -0.026 | -0.011 | 0.005 | 0.035 |
|  | ∆WD~SLA | -0.040 | -0.014 | -0.002 | 0.011 | 0.037 |
|  | ∆WD~H | -0.002 | 0.020 | 0.031 | 0.042 | 0.063 |
|  | ∆WD~SM | -0.050 | -0.024 | -0.011 | 0.003 | 0.029 |
|  | ∆WD~WD | 0.038 | 0.075 | 0.093 | 0.111 | 0.148 |
|  | HII~SLA | -0.005 | 0.010 | 0.017 | 0.025 | 0.041 |
|  | HII~H | -0.022 | -0.008 | -0.002 | 0.004 | 0.017 |
|  | HII~SM | -0.051 | -0.034 | -0.026 | -0.019 | -0.004 |
|  | HII~WD | -0.038 | -0.016 | -0.005 | 0.006 | 0.028 |
|  | SPEI~SLA | -0.033 | -0.014 | -0.005 | 0.004 | 0.022 |
|  | SPEI~H | -0.025 | -0.010 | -0.003 | 0.005 | 0.019 |
|  | SPEI~SM | -0.014 | 0.004 | 0.014 | 0.023 | 0.042 |
|  | SPEI~WD | -0.006 | 0.017 | 0.028 | 0.040 | 0.065 |
